# Mapping the Evolution of Digital Health Research: Bibliometric Overview of Research Hotspots, Trends, and Collaboration of Publications in JMIR (1999-2024)

**DOI:** 10.2196/58987

**Published:** 2024-10-17

**Authors:** Jing Hu, Chong Li, Yanlei Ge, Jingyi Yang, Siyi Zhu, Chengqi He

**Affiliations:** 1 Rehabilitation Medicine Center West China Hospital Sichuan University Chengdu China; 2 Key Laboratory of Rehabilitation Medicine in Sichuan Province West China Hospital Sichuan University Chengdu China; 3 School of Rehabilitation Sciences West China School of Medicine Sichuan University Chengdu China

**Keywords:** JMIR, bibliometric analysis, ehealth, digital health, medical informatics, health informatics, open science, publishing

## Abstract

**Background:**

While bibliometric studies of individual journals have been conducted, to the best of our knowledge, bibliometric mapping has not yet been utilized to analyze the literature published by the *Journal of Medical Internet Research* (*JMIR*).

**Objective:**

In celebration of the journal’s 25th anniversary, this study aimed to review the entire collection of *JMIR* publications from 1999 to 2024 and provide a comprehensive overview of the main publication characteristics.

**Methods:**

This study included papers published in *JMIR* during the 25-year period from 1999 to 2024. The data were analyzed using CiteSpace, VOSviewer, and the “Bibliometrix” package in R. Through descriptive bibliometrics, we examined the dynamics and trend patterns of *JMIR* literature production and identified the most prolific authors, papers, institutions, and countries. Bibliometric maps were used to visualize the content of published articles and to identify the most prominent research terms and topics, along with their evolution. A bibliometric network map was constructed to determine the hot research topics over the past 25 years.

**Results:**

This study revealed positive trends in literature production, with both the total number of publications and the average number of citations increasing over the years. And the global COVID-19 pandemic induced an explosive rise in the number of publications in *JMIR*. The most productive institutions were predominantly from the United States, which ranked highest in successful publications within the journal. The editor-in-chief of *JMIR* was identified as a pioneer in this field. The thematic analysis indicated that the most prolific topics aligned with the primary aims and scope of the journal. Currently and in the foreseeable future, the main themes of *JMIR* include “artificial intelligence,” “patient empowerment,” and “victimization.”

**Conclusions:**

This bibliometric study highlighted significant contributions to digital health by identifying key research trends, themes, influential authors, and collaborations. The findings underscore the necessity to enhance publications from developing countries, improve gender diversity among authors, and expand the range of research topics explored in the journal.

## Introduction

The *Journal of Medical Internet Research* (*JMIR*) is a renowned peer-reviewed journal that specializes in health informatics and digital health. It addresses a broad spectrum of topics, including emerging technologies, medical devices, apps, engineering, telehealth, and informatics applications for patient education, prevention, population health, and clinical care. Professor Gunther Eysenbach, a renowned expert in digital health and eHealth, established the journal *JMIR* in 1999 and has since served as its Editor-in-Chief. He has also defined digital health as health services and information delivered or enhanced through the internet and related technologies [1]. Because of its interdisciplinary nature, digital health spans various fields, including medical sciences, computer sciences, behavioral sciences, social sciences, communication sciences, psychology, library sciences, informatics, human-computer interaction studies, and related areas. To drive and accelerate the advancement of digital health, *JMIR* provides valuable insights across all these topics. The inaugural edition was released in August 1999, with a second publication later that same year. Beginning in 2022, *JMIR* has published 12 issues annually. Starting in 2023, the publications have been organized into annual volumes without individual issue numbers. Under JMIR Publications, Inc., 26 volumes have been published, with 1 volume released each year. Additionally, *JMIR* has curated 129 thematic collections to organize publications on specific digital health topics. A variety of metrics consistently place the journal among the top in its field. According to the SCImago Journal Rank powered by Scopus, *JMIR* is ranked in the top 25% (Q1) of the Medicine: Health Informatics discipline, with a ranking of 8th out of 123 (93rd percentile). Based on the 2023 Journal Citation Indicator from Clarivate, the journal ranks 5th out of 31 journals in the Medical Informatics category (85.5th percentile). In the Health Care Sciences and Services category, it is ranked 3rd out of 105 journals, placing it in the 97.6th percentile. Additionally, the journal has an impact factor of 7.4. Based on the scholarly influence of publications over the past 5 years, Google Scholar has ranked *JMIR* as the top journal in the field of Medical Informatics.

When a journal celebrates an anniversary or special occasion, it is customary to organize a specific academic undertaking, such as a review [2], an editorial [3], or a bibliometric study [4]. Computer-assisted statistics and software have significantly advanced the practice of bibliometrics. These powerful tools enable the summarization of journal contributions and cooperation patterns, as well as the analysis of large volumes of scholarly publications to understand the knowledge structure and developmental trends across various disciplines [5]. A retrospective bibliometric analysis of the European Journal of Innovation Management from 1998 to 2021 was conducted by Bamel et al [6]; Martorell Cunill et al [7] performed a bibliometric analysis for the International Journal of Hospitality Management’s 35th anniversary [7]; and Nath et al [8] examined publications in Community Dentistry and Oral Epidemiology from 1973 to 2022.

Given *JMIR*’s prominent position in the digital health sector, conducting a bibliometric analysis of this journal can provide valuable insights into the knowledge framework of the field. The readers, editors, and reviewers of *JMIR*, along with academics researching digital health, will gain a clear understanding of the current boundaries of the subject, as well as the key contributors, emerging topics, and evolving focal points.

To celebrate *JMIR*’s silver jubilee (25 years), this paper aims to retrospectively review the journal’s productivity, impact, emerging knowledge, and contributions to the digital health discipline since its establishment. This study analyzes the impact of the journal by examining its publication and citation patterns. It also identifies the most influential authors, institutions, and nations associated with the journal, along with an analysis of the authors’ keywords. To achieve these objectives, articles published in *JMIR* from its inception to the present were retrieved and analyzed. Three bibliometric analytical tools—CiteSpace, VOSviewer, and the R package Bibliometrix—were utilized to comprehensively assess the academic structure of *JMIR* publications. This study not only summarizes the established academic structure but also predicts potential future research focuses for *JMIR*.

## Methods

### Search Strategy

The publications spanning from January 1999 to February 2024 were obtained from the Web of Science Core Collection (WoSCC) database on February 22, 2024. The research used the strategies outlined in the source title and included articles and reviews. We excluded letters, revisions, proceedings papers, editorial materials, and retracted publications. The investigation was conducted independently by 2 researchers (JH and CL) to ensure the reliability and accuracy of the data collection process.

### Data Extraction and Analysis

The publications were collected and retrieved in the “Plain Text” format of “Full Records and References” from WoSCC. General information about *JMIR* publications was obtained from the WoSCC database. The bibliometric analysis of the included studies was conducted using CiteSpace 6.3 R1 Advanced, VOSviewer 1.6.19, and the R package Bibliometrix 4.1.2. This analysis focused on publications from January 1999 to December 2023. Publications from 2024 were discussed separately due to incomplete citation data, which could introduce bias into the analysis.

The software CiteSpace 6.3 R1 Advanced [9] was used to conduct a cocitation analysis, which included authors and references. Additionally, the software was used to create dual maps of journals and to discover citation bursts for keywords and authors. Clustering of keywords with labels generated from the keywords was performed to identify research themes and visualize the intellectual structure. Furthermore, a timeline view of keywords was created to visualize keyword evolution and identify trend shifts. The settings for CiteSpace were configured as follows: the period was set from January 1999 to December 2023, with each time slice representing a 1-year period. The strength of the linkages was measured using the cosine method, and the scope of the links was limited to each time slice. The selection criterion used was the g-index, with a value of *k*=25. Additionally, the network was pruned using the algorithm for pruning sliced networks and minimum spanning trees.

VOSviewer (version 1.6.19) [10] was used to analyze collaboration networks of countries, institutions, and authors through coauthorship analysis, with a minimum threshold of 5 documents per subject. VOSviewer also facilitated the analysis of keyword co-occurrence, using a minimum occurrence threshold of 5 keywords. The full count method was applied for all analyses. Network visualization, overlay visualization, and density visualization were used to depict the connections, temporal changes, and concentration of items within the network. The LinLog/modularity method was used to enhance the clarity and interpretability of the visualizations, facilitating a comprehensive understanding of collaboration patterns and keyword relationships. Additionally, the total link strength value was calculated for each analysis to measure connectivity and importance within the network [11].

The R package Bibliometrix (version 4.1.2) [12] was utilized to compute Lotka’s law [13] and analyze multiple-country publications (MCPs). The visualizations included collaborative globe maps of countries and trending subjects of keywords. Three-field plots were used to visualize the relationships and connections between countries, authors, and keywords, with the left field representing countries, the middle field representing authors, and the right field representing authors’ keywords. A thematic analysis of terminology was conducted based on “keyword plus” using a Walktrap clustering algorithm to identify the major research themes, their relative importance, interconnections, and evolving trends.

### Interpretation of Charts

#### Node

A node is defined as a single entity, such as a country, institution, author, or keyword. A connection between nodes signifies the presence of a co-occurrence relationship.

#### Betweenness Centrality

Betweenness centrality is a metric used to quantify the significance of nodes within a network. A higher betweenness centrality indicates greater connectivity relevance for the nodes [14].

#### Cluster View

A cluster view was created using the likelihood ratio statistic and keyword lists of cited articles within each cluster. The cluster name was automatically determined by selecting the keyword with the strongest correlation for each cluster. The effectiveness of the graph drawing was assessed using the modularity *Q* value and the weighted mean silhouette value (*S*). Clustering results were deemed satisfactory if the modularity *Q* was greater than 0.3 [15] and the homogeneity of the cluster structure was verified when the mean *S* was greater than 0.5 [14].

#### Dual-Map Overlap

The dual-map overlay visualizes the distribution and citations of works across different disciplines. The left panel displays the journals that are being cited, while the right panel presents the journals that cite them. The citation paths are indicated by colored stripes in the middle [16].

#### Burst Detection

Burst detection involves identifying changes in the frequency of keywords, which may occur when there is a significant shift in the number of citations.

## Results

### Global Trend in Publication Outputs and Citations

The study’s workflow, including the search strategy and method, is depicted in Multimedia Appendix 1. The search conducted using the WoSCC database resulted in a total of 7780 documents. After excluding 137 correlations, 2 proceeding papers, 71 editorial materials, 2 retracted publications, and 120 letters, 7448 publications were included in the data set. This data set involved the collaboration of 32,232 authors. Out of these publications, 6306 (84.67%) were categorized as articles, while 1142 (15.33%) were classified as reviews. General information about the papers is presented in Multimedia Appendix 2. The average annual publication count is 297.92. According to the “Web of Science Citation Report,” there have been a total of 226,105 citations for these articles, resulting in an average of 30.36 citations per article and 9044.2 citations annually.

**Figure 1:**
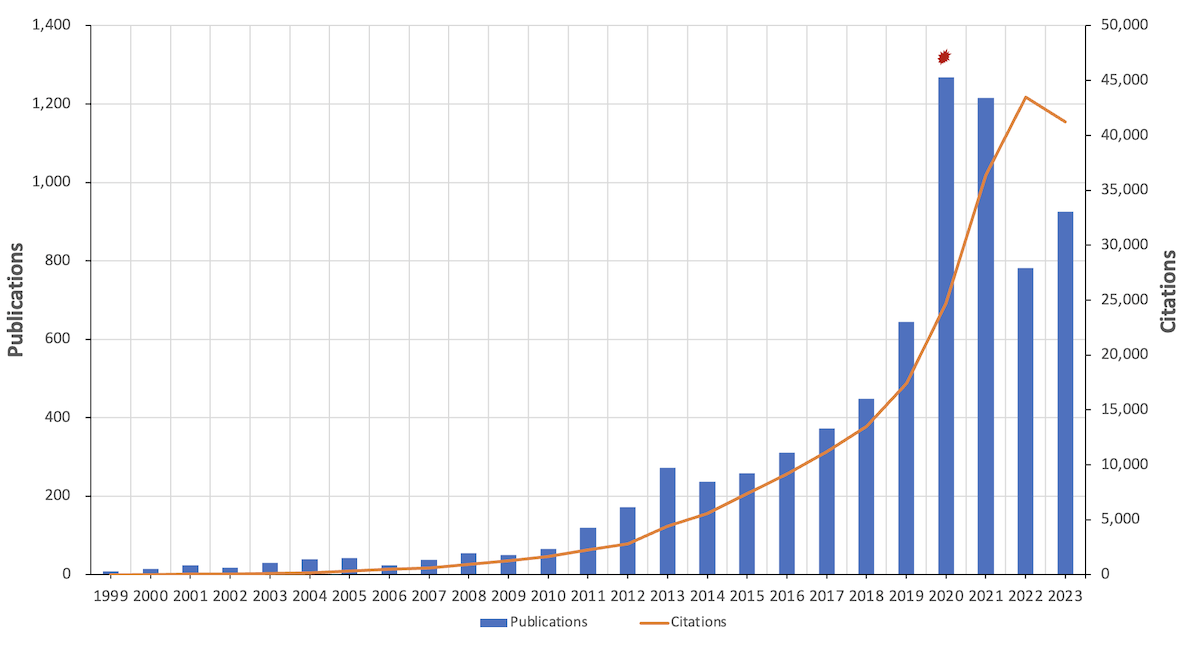
The number of annual publications (bar chart) and the number of annual citations (line chart) from 1999 to 2023 (source: the Web of Science Core Collection). The explosion symbol in the graphic signifies the significant increase in research activities during the global COVID-19 pandemic.

Figure 1 illustrates the patterns of yearly publications and citation values. The number of annual publications exhibited a consistent and gradual increase during the first 25 years of the century, followed by a sudden spike in 2022. The number of citations showed continuous and gradual growth until it reached a point of stability in the past 2 years. The cumulative H-index of *JMIR* is 177.

### Analysis of Authoritative Country/Region

**Figure 2:**
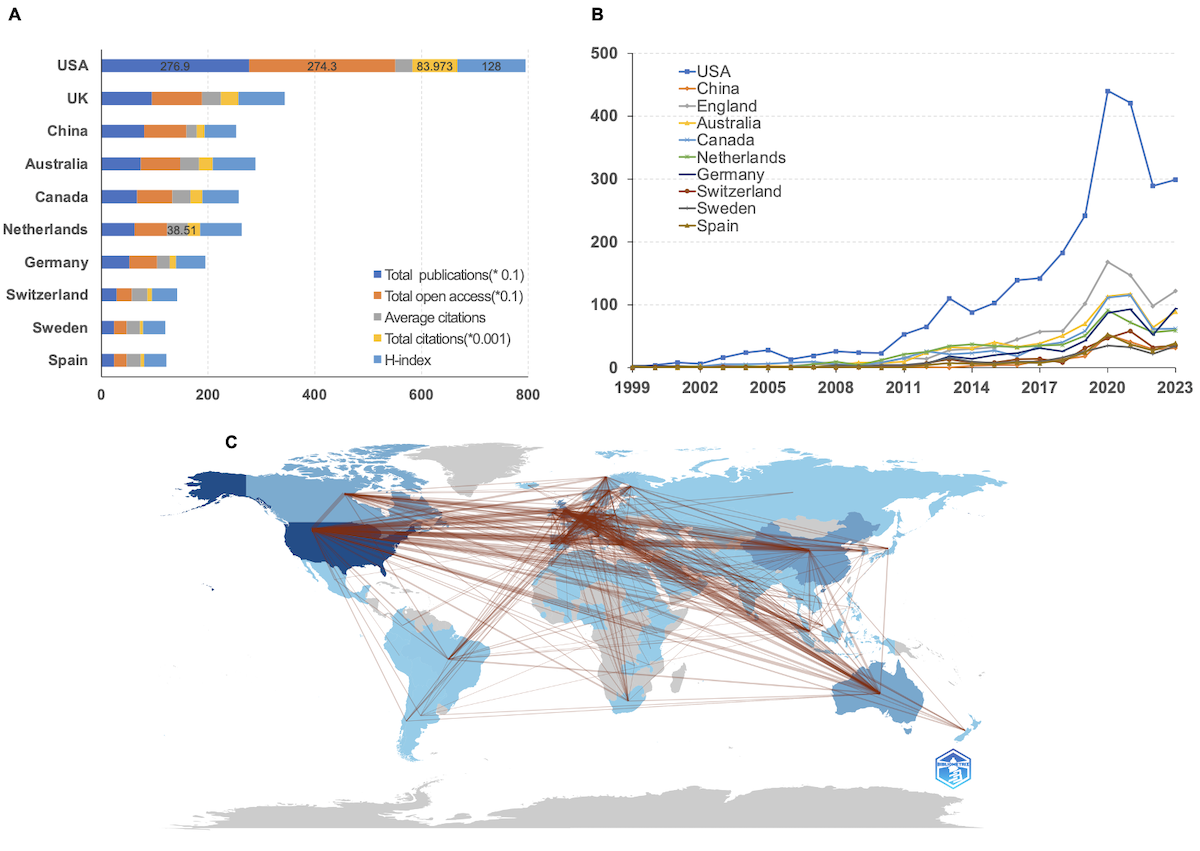
Analysis of countries/regions engaged in publications in the Journal of Medical Internet Research (JMIR). (A) Top 10 countries/regions with the largest number of publications (source: WoSCC); (B) top 10 countries/regions with the largest number of publications over time (source: WoSCC); (C) countries' collaboration world map (source: the R package Bibliometrix). WoSCC: Web of Science Core Collection.

A total of 120 countries contributed to publishing in *JMIR*. Table 1/Figure 2A presents the rankings of the 10 most prolific countries based on their total publication (TP). The United States ranked first with 2769 publications, accounting for 37.18% of the total 7448 publications. The United Kingdom followed with 949 publications, representing 12.74%, while China came in third with 798 publications, accounting for 10.71%. The United States stands out for having the highest number of publications and total citations (TC=83,973), along with an impressive H-index of 128. These figures reflect the country’s significant contributions to research in the field of digital health. Figure 2B displays the top 10 most productive nations, and the data suggest that the United States experienced a more notable rise in productivity compared with other countries. Outside the top 10 nations in terms of publications, the Netherlands had the highest number of citations per publication (average citation [AC]=38.51), followed by Australia (AC=35.65) and the United Kingdom (AC=35.25). Meanwhile, the United States had the most citations overall (AC=35.65; Table 1).

The ratio of MCPs measures the proportion of multiple-country publications among total publications, which highlights international cooperation in research. Among the top 10 most prolific nations, Switzerland (82/163, 50.3%), Spain (61/154, 39.6%), and Germany (148/384, 38.5%) have the highest ratios of MCPs for global collaboration, as shown in Multimedia Appendix 3. Although the United States (326/2156, 15.1%), the United Kingdom (197/619, 31.8%), and China (279/808, 34.5%) ranked as the top 3 nations in terms of the volume of publications involving multiple countries, their MCP ratios were not the highest due to the sheer volume of publications they have produced.

Based on the collaboration world map of countries (Figure 2C), the United States had the highest degree of collaboration with China, with a frequency of 230. This was followed by the United Kingdom, with a frequency of 166, and Canada, with a frequency of 146, as shown in Multimedia Appendix 4. Multiple countries have made significant contributions or engaged in collaboration with scholars in *JMIR* over varying durations. The United States, Canada, the Netherlands, and Australia have exhibited high levels of activity, with an average publication year of 2018. With advancements in computer and internet technologies, research in digital health is attracting increasing attention from scholars. Over the past 3 years, researchers from China, South Korea, and Brazil have demonstrated significant academic engagement and collaboration with other nations (Multimedia Appendix 5).

**Table 1:** Top 10 countries in terms of publications (source from WoSCC^a^).

Country	Total publication, n	Total open access, n	Average citations	Total citations, n	H-index
United States	2769	2743	31.79	83973	128
United Kingdom	949	940	35.25	32614	87
China	798	794	19.49	14955	59
Australia	740	733	35.65	25691	80
Canada	667	660	34.35	22494	68
The Netherlands	619	615	38.51	22870	78
Germany	524	522	23.27	11857	55
Switzerland	286	285	29.51	8280	47
Sweden	237	235	25	5793	42
Spain	236	235	26.73	6225	42

^a^WoSCC: WoSCC: Web of Science Core Collection.

### Analysis of Publishing Institution

**Figure 3:**
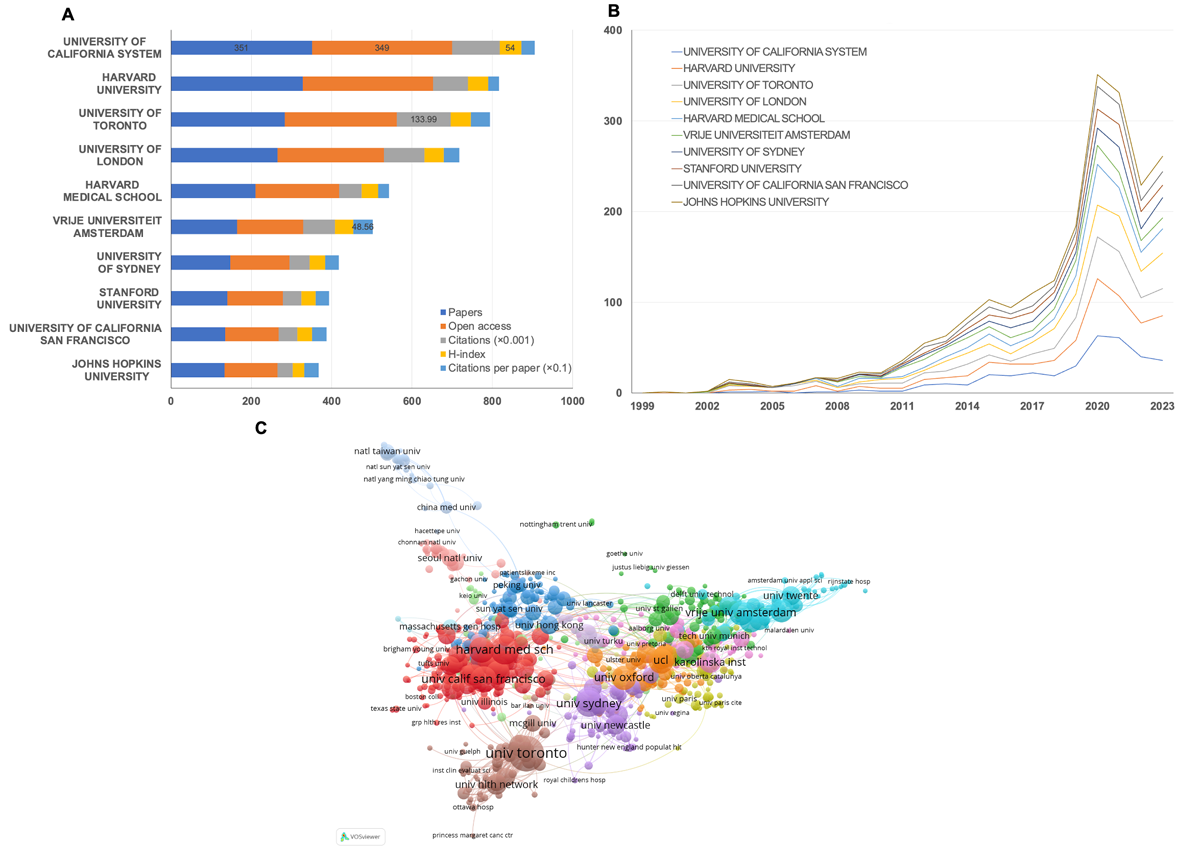
Analysis of institutions engaged in publications in the Journal of Medical Internet Research (JMIR). (A) Top 10 institutions with the largest number of publications (source: WoSCC); (B) top 10 institutions with the largest number of publications over time (source: WoSCC); (C) institutions' collaboration network (source: VOSviewer). WoSCC: Web of Science Core Collection.

A total of 6689 institutions contributed to publications in *JMIR*. Figure 3A displays the top 10 institutions based on the number of papers. The 3 leading institutions are the University of California System, with 351 publications (4.71%); Harvard University, with 328 publications (4.40%); and the University of Toronto, with 283 publications (3.80%). The trend in institutions’ contributions to *JMIR*, in terms of the number of research articles published over time, has remained similar across institutions, as shown in Figure 3B. The level of collaboration among institutions is substantial, as depicted in Figure 3C. The University of Toronto had the highest level of collaboration with several institutions, including the University Health Network Toronto (link strength=78), Women’s College Hospital (link strength=31), and the Centre for Addiction and Mental Health (link strength=28). Meanwhile, Harvard Medical School and Harvard University engage in extensive relationships with other scholarly institutions, as indicated in Multimedia Appendix 6. Multimedia Appendix 7 displays a timeline view of institutional collaboration. Harvard Medical School, one of the most productive institutions, has recently been active in *JMIR*, with an average publication year of 2020. Several scholarly institutions in Asia have also been actively involved in and made significant contributions to *JMIR* in recent years, including Peking University, Sun Yat-sen University, and National Taiwan University.

### Analysis of Author Distribution

**Figure 4:**
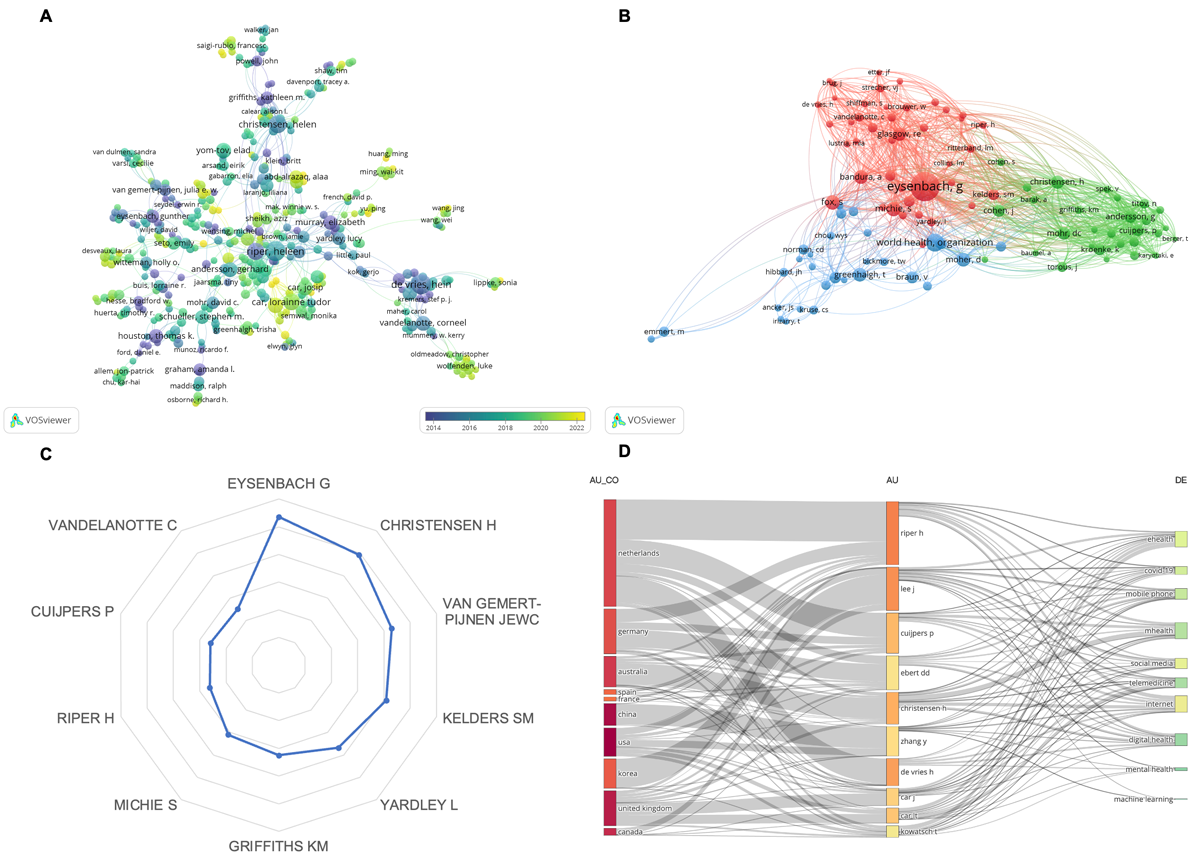
Analysis of authors engaged in publications in the Journal of Medical Internet Research (JMIR). (A) Coauthorship network of authors (source: VOSviewer); (B) cocitation network of authors (source: VOSviewer); (C) authors' local citation map (source: the R package Bibliometrix); (D) the 3-field plot—middle field: authors; left field: country; right field: keywords (source: the R package Bibliometrix).

The authors demonstrating the highest levels of productivity were Heleen Riper (TP=41) from Vrije Universiteit Amsterdam, Hein de Vries (TP=39) from Maastricht University, and Jinseok Lee (TP=39) from Kyung Hee University. Data regarding the top 10 authors with the highest publication frequency were retrieved from the WoSCC database and are presented in Multimedia Appendix 8. Three out of the 10 most prolific authors hailed from the Netherlands. Additionally, an analysis was conducted on the worldwide academic influence of *JMIR* authors. Multimedia Appendix 9 displays information on the top 25 influential writers based on the strength of citation bursts. Gunther Eysenbach had the most significant burst in citations since 1999, with a strength of 11 from 1999 to 2011. Hein De Vries had the highest burst strength of 12.29 from 2012 to 2017. Tobias Kowatsch (strength=8.11, 2021-2024) and Felix Balzer (strength=3.76, 2020-2024) were the most recent authors with strong citation bursts. Figure 4A and Multimedia Appendix 10 display the author’s coauthorship network mapping and its strength. Heleen Riper collaborated extensively with other authors, demonstrating a total link strength of 100. The average publication year of their work was 2016. Hein de Vries and Lorainne Tudor Car, both affiliated with Nanyang Technological University, also have significant scholarly connections with other authors. Hein de Vries has a total link strength of 81, while Lorainne Tudor Car has a total link strength of 76. Additionally, Lorainne Tudor Car has remained academically active, with an average publication year of 2020. Cocitation analysis identified Gunther Eysenbach, Helen Christensen, and Gerhard Andersson as the 3 most frequently cited authors in connection with other *JMIR* authors (Figure 4B/Multimedia Appendix 11). The most significant cocitation link emerged between Gunther Eysenbach and Susannah Fox, with a link strength of 355. According to the authors’ local citation map (Figure 4C), Gunther Eysenbach (local citations=536), Helen Christensen (local citations=493), and Julia EWC van Gemert (local citations=429) were the most frequently cited by other *JMIR* authors. A 3-field map shown in Figure 4D helps visualize the links between the authors, nations, and keywords. Most authors from the Netherlands who have made exceptional contributions to publishing in *JMIR* focus on themes such as eHealth, COVID-19 impacts, and mobile phone apps. Lotka’s law was assessed by quantifying the authors’ publication frequency in *JMIR* as a measure of their productivity (Multimedia Appendix 12). The data revealed that 77.54% (24,993/32,232) of the authors contributed to *JMIR* publications by submitting at least one paper, while 13.45% (4336/32,232) published a second article.

### Analysis of Journal Distribution

**Figure 5:**
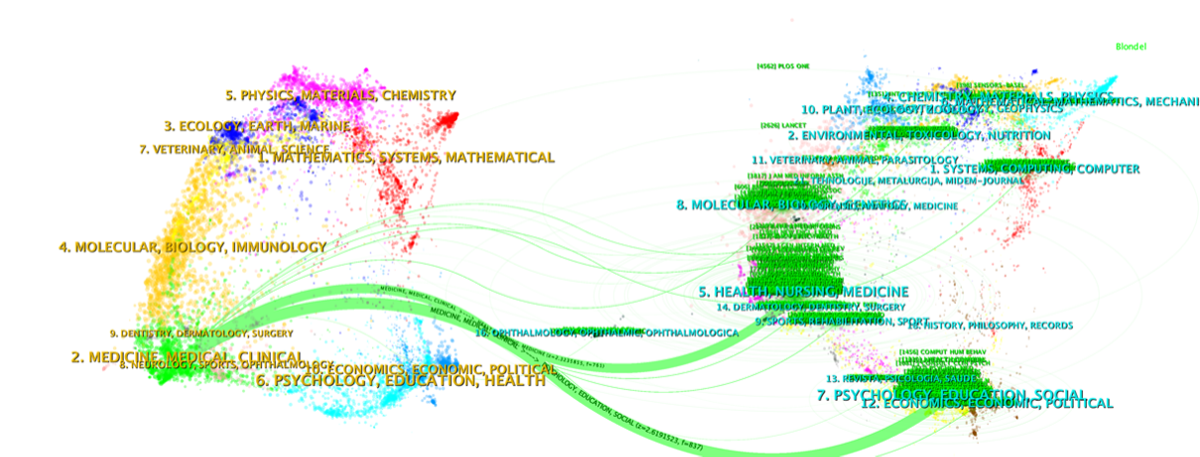
The dual‐map overlay of journals in the field of frailty and depression research (source: CiteSpace).

Our investigation revealed that a total of 11,896 journals were referenced by publications in *JMIR*. Multimedia Appendix 13 presents a concise overview of the 25 most frequently cited publications in *JMIR*. In addition to *JMIR* itself (n=5982), the most commonly cited academic journals include the International Journal of Environmental Research and Public Health (n=2538), PLoS One (n=1915), and JMIR mHealth and uHealth (n=1641). In terms of the distribution of articles citing *JMIR* publications, the majority of citations originated from *JMIR* itself (n=24,714), followed by PLoS One (n=4562) and the Journal of the American Medical Informatics Association (n=3877). Multimedia Appendix 14 provides additional frequently cited sources. According to data from the Web of Science, most articles citing *JMIR* publications can be classified into the categories of Health Care Sciences Services (21,539/120,399, 17.89%, articles citing *JMIR* publications), Medical Informatics (17,144/120,399, 14.24%), and Public Environmental Occupational Health (17,072/120,399, 14.18%). The dual-map overlay in Figure 5 illustrates the distribution of topics among journals. This overlay consists of 2 sides: the citation channels indicate that research published in health, nursing, medicine, psychology, education, and social journals is frequently cited by studies published in *JMIR*, which falls under the category of medicine, medical, and clinical journals.

### Analysis of Highly Impact Publications

Table 2 highlights the 10 most widely referenced publications from *JMIR*, which have had a significant academic influence on a global scale. Among these, 5 are reviews and 5 are original articles. Four of the publications report on randomized controlled trials [17-20]. Three publications explored the topic of mental health [19-21]. Additionally, the significant role of compliance in digital health care is discussed in 5 out of the 10 studies [17-20,22]. An article published in 2005, which examined attrition in eHealth trials [22], has the highest number of citations (TC=1615), averaging approximately 80.75 citations per year. The most recent publication among the top 10 notable articles is by Andras Lanczky et al [23], which describes a web-based tool for conducting univariate and multivariate Cox proportional hazards survival analyses using data from transcriptomic, proteomic, metabolomic, or genomic investigations. This publication had the highest citation density of 182.5 among all the publications. Another notable citation density was found in a review examining the impact of the COVID-19 epidemic on the mental health of college students, which had a citation density of 180 [21]. Multimedia Appendix 15 (see also [17-19,22,24-29]) provides a summary of the 10 most frequently cited publications by local authors. The intellectual foundation of *JMIR* is further enriched by publications from other sources, with data regarding the top 10 references available in Multimedia Appendix 16 (see also [17,18,22,30-36]). Among the 10 highly cited articles, 5 were published in *JMIR*, and the authors predominantly referenced statements related to systematic reviews and meta-analyses [30].

**Table 2:** The top 10 high-impact articles published between 1999 and 2023 (sourced from the Web of Science Core Collection).

No	Reference	Type	First author, year	Last author	Main find	Total citations, n	Country	Duration (years)	Citation density
1	[22]	Article	Gunther Eysenbach, 2005	Single author	This article advocates for the necessity of a “science of attrition,” which entails the development of models to understand the termination of eHealth apps and the occurrence of participants dropping out of eHealth trials.	1615	Canada	20	80.75
2	[17]	Review	Thomas L. Webb,2010	Susan Michie	This review assessed theory and behavior change strategies to determine the best internet-based treatments to promote health behavior change. A new scoring scheme was also created for measuring internet-based intervention modes of delivery and linking modes to effect sizes.	1582	England	15	105.47
3	[24]	Review	Anne Moorhead, 2013	Ciska Hoving	Social media is a strong tool for collaboration and social engagement. Social media for health communication has many benefits, but the information provided must be checked for quality and dependability and users’ confidentiality and privacy must be safeguarded.	1264	North Ireland	12	105.33
4	[25]	Article	Cameron D. Norman, 2006	Harvey A. Skinner	A model of eHealth literacy was presented, covering the abilities customers need to profit directly from eHealth. Health practitioners can use a resource list and a literacy-type profile with examples of patient-client issues to increase literacy across each domain.	1232	Canada	19	64.84
5	[21]	Article	Changwon Son, 2020	Farzan Sasangohao	The COVID-19 pandemic introduced increased levels of stress, anxiety, and depression in higher education.	900	The United States	5	180
6	[37]	Article	Trisha Greenhalgh, 2017	Sara Shaw	A Nonadopting, Abandonment, Scale-Up, Spread, and Sustainability (NASSS) framework can help predict and evaluate the success of a technology-supported health or social care program.	871	The United Kingdom	8	108.88
7	[18]	Review	Saskia M. Kelders, 2012	Julia E. W. C. an Gemert-Pijnen	Although health care settings differ in intervention characteristics, they do not predict adherence. Technology and interaction differences predict adherence.	765	The Netherlands	13	58.85
8	[19]	Review	Helen Christensen, 2009	Louise Farrer	Randomized controlled trials of web interventions have lower dropout rates than open access websites. Internet intervention research and behavioral health literature both require theoretical models of adherence. Disease-related anxiety and sadness need more research.	749	Australia	16	46.81
9	[23]	Article	Andras Lanczky, 2012	Balazs Gyorffy	This study introduced a web-based tool capable of performing univariate and multivariate Cox proportional hazards survival analyses using data generated by genomic, transcriptomic, proteomic, or metabolomic studies.	730	Hungary	4	182.5
10	[20]	Review	Tara Donker, 2013	Helen Christensen	Apps for mental health have the potential to be very beneficial and increase treatment accessibility. The few evidence-based mental health apps that are now in the public domain need to be identified, and the public has to be made aware of this.	696	Australia	12	58.0

### Analysis of Keywords Co-occurrence

**Figure 6:**
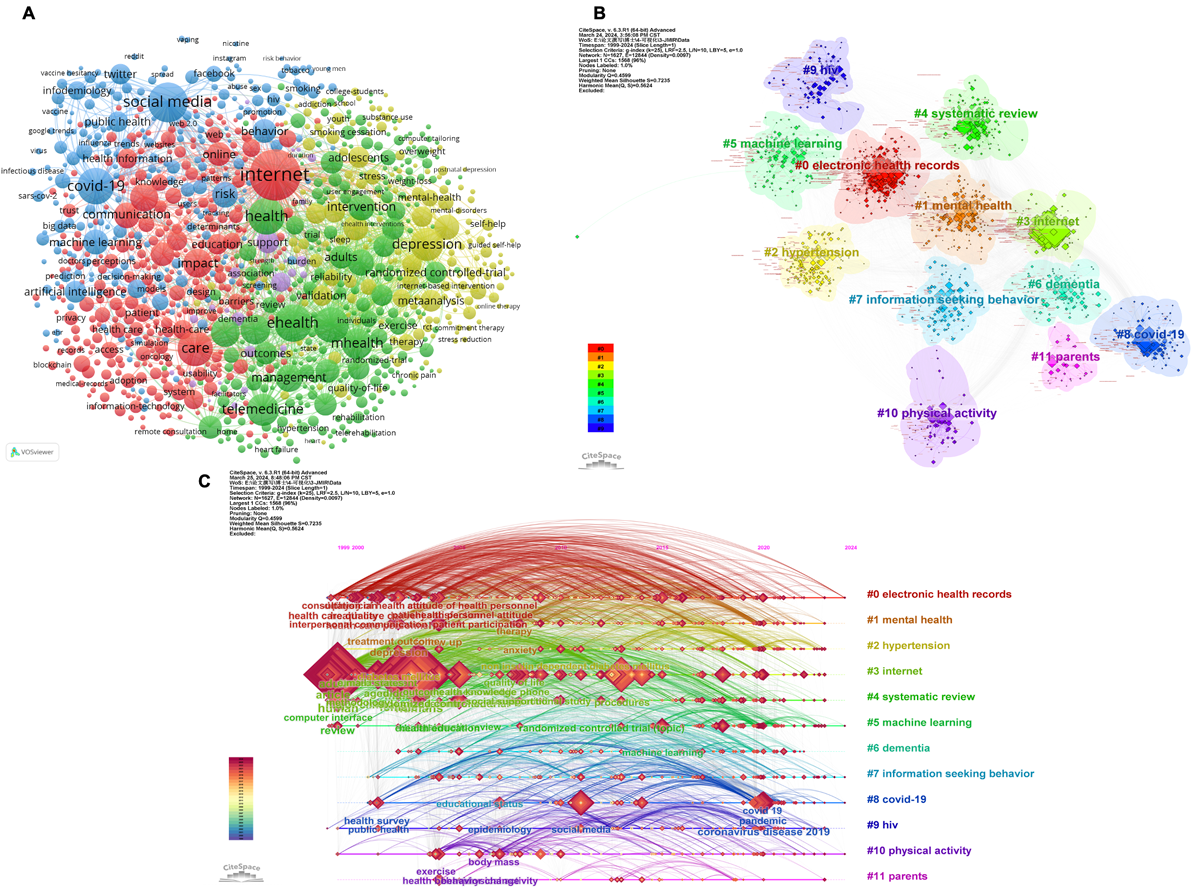
Analysis of keywords of Journal of Medical Internet Research (JMIR) publications: (A) keyword co-occurrence network (source: VOSviewer); (B) keyword cluster analysis (source: CiteSpace); (C) timeline view of keywords (source: CiteSpace).

Keyword co-occurrence analysis is a powerful bibliometric tool that accurately identifies current issues within the scientific knowledge structure. We conducted a co-occurrence analysis of keywords in *JMIR* by examining the frequency of their occurrences (Figure 6A). The 5 most frequently used keywords were internet (n=1524), eHealth (n=810), social media (n=904), COVID-19 (n=784), and health (n=770). The term “quality” exhibited the highest degree of centrality, with a value of 1.11. Additionally, a total of 12 distinct clusters were generated using the log-likelihood ratio to examine keyword co-occurrence (Figure 6B and Multimedia Appendix 17). The largest cluster (#0), designated “electronic health records,” comprised 321 members and had a silhouette value of 0.566. The most frequently highlighted members of this cluster included patient portals and electronic health records. The second cluster, classified as “mental health,” boasted a silhouette value of 0.67 and consisted of 181 members, with mental health, depression, and anxiety being the most commonly mentioned topics. The third cluster (#2), named “hypertension,” comprised 152 nodes and had a silhouette value of 0.722. The 3 most frequently mentioned terms in this cluster were hypertension, diabetes, and medication adherence. The investigation of cluster #0, which focuses on “electronic health records,” was initiated by the authors of *JMIR* in 1999 and has continued to the present day. After clustering, the timeline view of the keywords was analyzed and is shown in Figure 6C. The size of the nodes is directly proportional to the frequency of keyword occurrences. The color of the clusters indicates the evolution of research emphasis over time, with the most recently established connections represented in red or yellow and positioned at the top of the image. Initially, a significant number of researchers focused on the topic of the internet, followed by numerous studies examining diabetes and COVID-19. However, over the past 2 years, research has increasingly concentrated on electronic medical records and hypertension.

### Changes in Trends of Research

**Figure 7:**
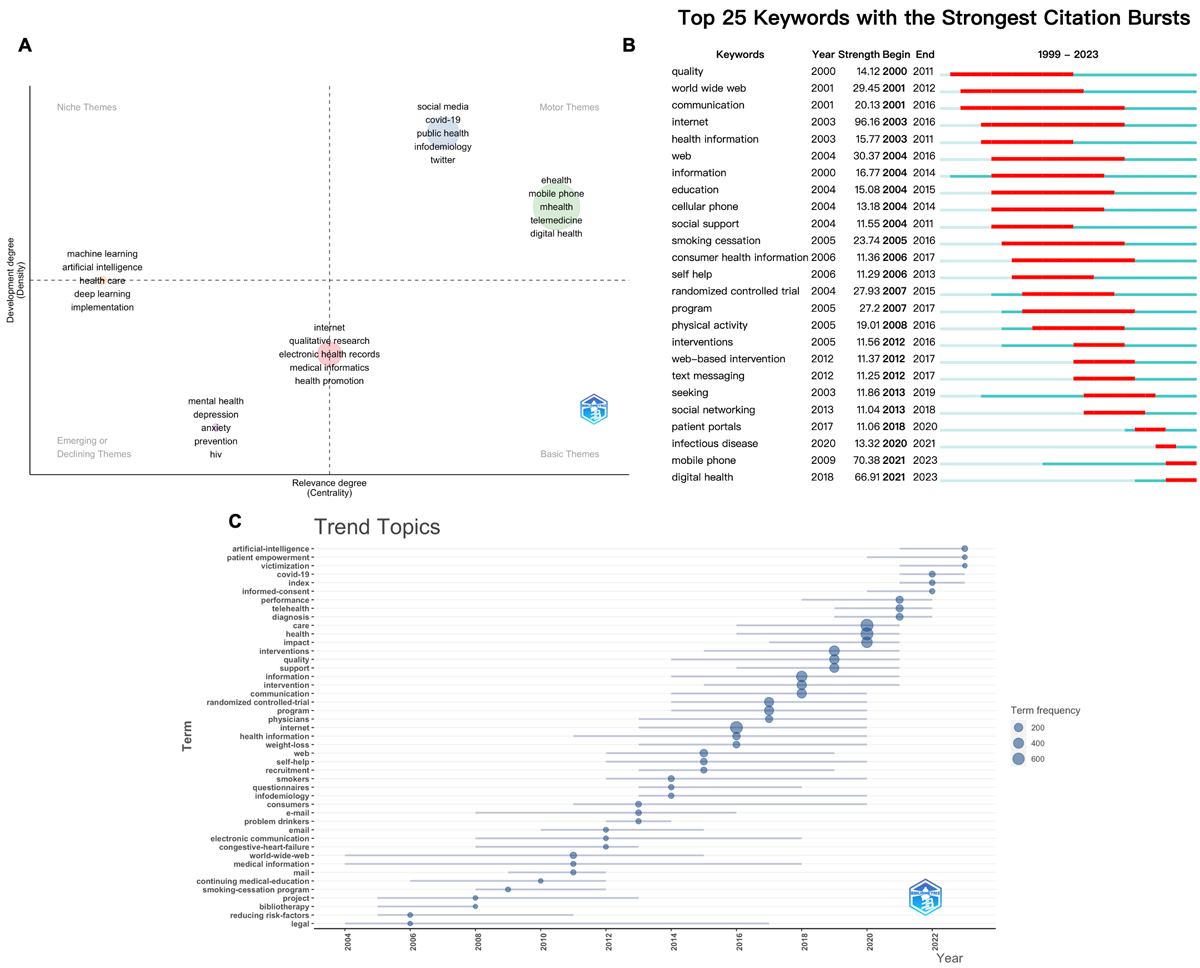
Analysis of keywords engaged in frailty and depression research. (A) thematic terminology analysis of keywords (source: the R package Bibliometrix); (B) the top 15 keywords with the strongest citation bursts (source: CiteSpace); (C) Trend topics of keywords (source: the R package Bibliometrix).

The study conducted a thematic terminology analysis (Figure 7A) to examine the main issues present in *JMIR* publications. The x-axis represents the centrality of network clusters, indicating the degree of connectivity with other clusters in the graph, which reflects the significance of a research topic. The y-axis represents density, quantifying the internal robustness and growth of a cluster network’s subject [38]. Clusters in the motor themes exhibit high centrality and density, suggesting that these topics are well-developed and essential for structuring research. Currently, the fields of digital health—specifically through social media and mobile phones—and public health, including infodemiology and COVID-19, are positioned in the motor themes quadrant. This indicates that extensive studies have been conducted on these issues, which are directly linked to the central theme of *JMIR*. Themes located in the emerging or declining quadrants exhibit both low centrality and low density, indicating that they are not well-developed and are on the margins. Investigations and preventive efforts targeting HIV and mental health issues, such as depression and anxiety, were situated in the third quadrant, suggesting that research in these areas is still in its nascent phase. Meanwhile, research on the application of artificial intelligence (AI)—including deep learning and machine learning—was positioned in the space between the first and third quadrants, indicating that while it remains in its early stages, it has a strong connection to the significant domain of digital health. Research on electronic health records and health informatics, positioned within the basic themes, has demonstrated that these areas require collaborative research across multiple disciplines [39]. Figure 7B illustrates the top 25 keywords with the strongest citation bursts. The keyword “quality” experienced the most significant increase in citations since 1999, with a burst strength of 14.12 from 2000 to 2011. From 2003 to 2016, “internet” exhibited the highest burst strength at 96.16. The latest trending keywords include “mobile phone” (n=70.38, 2021-2023) and “digital health” (n=66.91, 2021-2023). Additionally, the examination of trending topics revealed that “artificial intelligence,” “patient empowerment,” and “victimization” are emerging trends within this field (Figure 7C).

### The Most Recently Published Articles in JMIR

To monitor the publishing patterns in *JMIR*, we conducted a search and analysis of recently published articles from 2024. Eight publications from *JMIR* were collected during the literature retrieval on February 22, 2024. A 3-field plot illustrated insights regarding the authors, countries, and keywords associated with these newly published articles (Multimedia Appendix 18). Among these, 5 papers focused on the implementation of telehealth programs in various contexts [40-44]. Jiang et al [40] and Bazzano et al [44] investigated chronic health conditions, while Chang et al [41] and Chen et al [43] focused on the impact of telehealth on cardiovascular health. In addition to examining the application of telehealth programs, Jiang et al [40] and Lim et al [45] researched the development and deployment of conversational agents. These software programs significantly enhance communication in health care, improving its overall effectiveness. The recently published articles also provided valuable insights for stakeholders on efficiently managing income and expenditures while optimizing telehealth initiatives [41,42,44].

## Discussion

### Principal Findings

To commemorate the *JMIR*’s 25th anniversary of publication in 2024, we conducted an advanced bibliometric study. The objective of this study was to analyze the bibliometric characteristics of the journal from 1999 to 2024. This involved investigating patterns and changes in research literature production, as well as identifying the most productive countries, institutions, authors, research terms, and topics, along with their evolution over time. Furthermore, we were interested in identifying emerging subjects through keyword analysis and trend tracking.

Our descriptive bibliometric analysis revealed that *JMIR* primarily focused on original articles (6306/7448, 84.67%; Multimedia Appendix 2) and review articles (1142/7448, 15.33%; Multimedia Appendix 2) related to health services and digital health. Before 2010, the journal published approximately the same number of articles each year. However, beginning in 2011, there was a significant increase in annual publication volume, which continued through 2023. *JMIR*’s annual publication volume peaked in 2022 and continued to rise into 2023 (n=309.75). The slight decrease in the annual number of articles from 2021 to 2024 may be attributed to the emergence of COVID-19 and the subsequent economic downturn. Additionally, *JMIR*’s increasing emphasis on publishing higher-quality articles could contribute to this trend. The mean number of citations per article was 30.36, significantly exceeding the journal’s impact factor (7.4). This suggests that the articles published in *JMIR* are of high quality and have been extensively referenced by scholars worldwide. The correlation between the number of citations and the number of annual publications indicates that the journal consistently produces high-quality papers. Consequently, the volume of articles published has a substantial impact on the annual citation count.

In terms of the volume of articles published and the number of active institutions, the majority of contributing countries are developed nations with advanced levels of ecological and technological development. The United States has made the most significant contribution to *JMIR*, accounting for approximately 37.18% (2769/7448; Table 1) of all publications. Furthermore, 4 institutions based in the United States are among the top 10 most productive institutions. In terms of national productivity, the United Kingdom and China ranked second and third, respectively, with contributions of 12.74% (949/7448) and 10.71% (798/7448). Two institutions from the United Kingdom were included in the top 10 most productive institutions, while China had no relevant institutions in this category. Despite the presence of numerous research institutions in China, these findings suggest that there has been insufficient emphasis on digital health. The investigation also revealed that these publications were biased toward industrialized, English-speaking nations, potentially limiting the journal’s influence and applicability for practitioners and scholars in other regions. In terms of article units, Switzerland (82/163, 50.3%), Spain (61/154, 39.6%), and Germany (148/384, 38.5%) had the highest MCP ratios (Multimedia Appendix 3), significantly surpassing that of the United States (326/2156, 15.1%). This suggests that these 3 countries were more engaged in multi-institutional collaboration. The national collaboration map indicated that the United States and China had the closest cooperation, with a frequency of 230, suggesting that the United States should enhance its multicountry exchanges and collaborative efforts.

Heleen Riper, a researcher from Vrije Universiteit Amsterdam, was the most productive author and collaborated extensively with other writers. However, the strongest cocitation link was identified between Gunther Eysenbach and Susannah Fox, with a link strength of 355. Notably, Gunther Eysenbach experienced the most significant increase in citations between 1999 and 2011, achieving a strength score of 11. Tobias Kowatsch is currently the most active author, with a strength score of 8.11 between 2021 and 2024. According to the authors’ local citation map, Gunther Eysenbach is identified as the most influential author, with a total of 536 local citations. These results indicate that Eysenbach has significant influence in this domain and is dedicated to fostering international exchanges and collaborations in the digital health and health care sectors. As the editor-in-chief of *JMIR*, Gunther Eysenbach is affiliated with the University of Victoria. His research interests primarily revolve around health care, with a specific focus on health policy, eHealth, and consumer health informatics [22,46-49]. The analysis of the 3-field plot revealed that the most prominent authors originated from the Netherlands. These researchers mainly concentrated on eHealth [50-53], particularly emphasizing its impact on mental health [54-57] and the application of mobile phones [51,58] in their studies. Multiple analyses indicate that women authors remain inadequately represented, and this gender disparity may be attributed to unconscious bias in the peer review process, as well as insufficient empowerment and exploration of women’s contributions in academia. This underscores the need for additional support for significant female authors in the future [59,60].

The top 10 globally cited publications (Table 2) and locally cited publications (Multimedia Appendix 15) from *JMIR* were summarized. Local citations refer to references from within *JMIR*, while global citations encompass citations across the entire academic literature. Local citations evaluate impact within a specific field, whereas global citations assess overall influence across disciplines [61]. The overlap between these 2 lists highlights the significance and relevance of certain publications within both the broader academic community and the specific field addressed by *JMIR*. For instance, the article titled “The Law of Attrition,” published by editor Gunther Eysenbach in 2005 [22], has been cited 1615 times (WoSCC), ranking at the top of both lists. It emphasizes the importance of establishing a “science of consumption” and developing models to understand the termination of eHealth apps and the withdrawal of participants from eHealth trials. This work was essential in advancing and establishing the eHealth model. A subsequent review conducted in 2010 evaluated various theoretical and behavioral change methodologies to identify the most effective internet-based treatments for promoting healthy behavioral change [17]. A novel scoring system has been developed to assess internet-based interventions and efficient connection models [37]. This framework serves as a guide for the future implementation of internet-based therapies. In 2020, Son et al [21] published a study titled “Effects of COVID-19 on College Students’ Mental Health in the United States: Interview Survey Study,” which highlighted the importance of prioritizing the mental well-being of students affected by the pandemic [21]. Further analysis revealed that the papers with the highest citations among the top 10 primarily focused on mental health, internet-based tools, and eHealth.

Keywords serve as valuable tools for identifying core themes, primary study subjects, and content within a specific domain. Based on the co-occurrence analysis conducted using VOSviewer, the themes most extensively researched in *JMIR* were “internet” (n=1524), “eHealth” (n=901), and “social media” (n=810). Additionally, the analysis revealed 4 clusters, each represented by different colors. “Internet” emerged as the keyword with the highest frequency and was most commonly associated with other keywords. COVID-19 was the most prominent disease of concern in this journal and demonstrated the strongest connection with “social media.” This finding aligns with the perspective that COVID-19 has accelerated the development and adoption of digital technologies, including information dissemination, health communication, data collection, community support, behavioral insights, and the promotion of digital health tools [62,63]. *JMIR* is particularly focused on how public health crises impact human health and the potential benefits of digital health. Since the onset of COVID-19, *JMIR* has published 3 themes related to the epidemic. According to the clustering results from CiteSpace, the prominent keyword clusters included “electronic health records,” “mental health,” and “internet.” However, *JMIR* has also featured articles addressing “hypertension,” “dementia,” and “HIV.” The silhouette scores for all clusters were greater than 0.5, indicating highly credible clusters [64]. To remain relevant and impactful in the rapidly expanding field of digital health, *JMIR* may need to broaden the range of subjects it investigates. Based on the thematic terminology analysis of keywords from the R package Bibliometrix, the themes of “machine learning,” “health care,” and “implementation” were found to be underdeveloped and marginalized. These themes represent specific research areas that have achieved a certain level of maturity but still require significant contributions to the field. By contrast, the research themes of “depression,” “anxiety,” “prevention,” and “HIV” are either experiencing notable advancements or a decline in research interest. The COVID-19 pandemic and HIV significantly impact global health, with anxiety and despair closely associated with these crises [65,66]. An examination of keywords has shown that the emergence of digital health, driven by the COVID-19 pandemic, has garnered significant attention in recent years (2021-2023).

The current and future key focal points of *JMIR* include “artificial intelligence,” “patient empowerment,” and “victimization.” With the advancement of computer science and technology, AI is praised as a possible agent of change that will revolutionize the field of medicine as well as digital health. *JMIR* has published 7 AI-related themes, encompassing areas such as communication, detection, diagnosis, treatment, and analysis. It has been observed that AI’s capabilities significantly enhance diagnostic accuracy, personalize treatment, improve operational efficiency, and support clinical decision-making. These advancements in digital health are leading to improved patient outcomes, enhanced care delivery, and increased operational efficiencies within the health care system [67,68]. Furthermore, digital health technologies play a pivotal role in enhancing patient empowerment by providing tools and resources that enable patients to actively manage their health [69]. AI further promotes patient engagement and empowerment by delivering individualized health services, including education, decision-making aids, and interactive support. The contribution of digital health to patient empowerment is an area of growing interest for *JMIR*, as evidenced by the recent publication of 122 articles utilizing this keyword. Another trending topic is “victimization” with *JMIR* recently publishing 67 related articles. Risks in both daily and digital environments underscore the importance of patient empowerment. *JMIR* integrates digital health into support systems for persons who experienced violence to enhance the accessibility, effectiveness, and safety of digital health services. There is a growing concern and interest in these topics, reflecting their increasing relevance in the field of digital health.

We propose several strategies to enhance the impact and relevance of *JMIR*: (1) advocating for the adoption of novel and advanced research methods, along with cutting-edge statistical techniques for data analysis, such as data linkage, machine learning, and AI; (2) fostering journal submissions by establishing partnerships with universities and research institutions, or by creating country-specific special issues to broaden the journal’s international scope and include articles from a more diverse range of geographical locations; (3) promoting author diversity to ensure a wider representation of ideas and perspectives in the journal; and (4) prioritizing research on topics that address emerging themes and encourage cross-disciplinary investigations.

### Limitations

This study uses a bibliometric approach to comprehensively examine the *JMIR* literature published between 1999 and 2024. However, this study has certain limitations. All publication data were obtained from the WoSCC, which means that some publications may have been inadvertently overlooked. Additionally, the accuracy of the findings may be affected when converting the data format to merge documents from multiple databases, as each database may have distinct characteristics. Nonetheless, the WoSCC database is considered the most representative, as it encompasses a majority of the prominent and essential scholarly journals worldwide. Therefore, we assert that the current research findings accurately reflect the global state of this discipline. Another unavoidable issue is the lack of transparency in the algorithms used by bibliometric software, which may lead to algorithmic bias. To address this concern, 3 distinct bibliometric analysis software programs were utilized to generate a comprehensive set of information.

### Conclusions

A bibliometric analysis conducted from 1999 to 2024 found that *JMIR* published a total of 7780 articles authored by 32,232 individuals. The journal featured a diverse group of authors from various continents; however, the United States emerged as the most prolific country, with its institutions making significant contributions. The number of articles published in *JMIR* increased steadily, with a notable spike in 2022. The highest average number of publications, 309.75, was recorded in 2023. The study also identifies the most productive and innovative subjects published in *JMIR*, highlighting promising avenues for future research. Additionally, this analysis provides valuable insights into the journal’s history and current standing, offering readers and potential contributors essential information for future development.

## Appendix

Multimedia Appendix 1 Flowchart of bibliometric analysis.

Multimedia Appendix 2 A descriptive summary of JMIR publications from 1999 to 2024 (source: the R package Bibliometrix).

Multimedia Appendix 3 Corresponding author’s countries (source: the R package Bibliometrix).

Multimedia Appendix 4 The top 25 countries' collaboration (source: the R package Bibliometrix).

Multimedia Appendix 5 The timeline view of coauthorship analysis network of country (source: VOSviewer).

Multimedia Appendix 6 Collaborations among scholar institutions (source: VOSviewer).

Multimedia Appendix 7 The timeline view of coauthorship analysis network of institutions (source: VOSviewer).

Multimedia Appendix 8 The top 10 most frequently published authors (source: Web of Science Core Collection).

Multimedia Appendix 9 The top 25 authors with the strongest citation bursts (source: CiteSpace).

Multimedia Appendix 10 The top 10 most frequently collaborated authors (source: VOSviewer).

Multimedia Appendix 11 The top 10 influential authors among cocitation analysis (source: VOSviewer).

Multimedia Appendix 12 Author’s productivity through Lotka’s law (source: the R package Bibliometrix).

Multimedia Appendix 13 The top 25 most citing sources by the JMIR authors (source: Web of Science Core Collection).

Multimedia Appendix 14 The top 10 most locally cited sources (source: the R package Bibliometrix).

Multimedia Appendix 15 The top 10 most locally cited documents (source: the R package Bibliometrix).

Multimedia Appendix 16 The top 10 citing references by the JMIR authors (source: Web of Science Core Collection).

Multimedia Appendix 17 Summary of 11 clusters analysis for keywords (source: CiteSpace).

Multimedia Appendix 18 The 3-field plot graph of the 8 publications in 2024 (source: the R package Bibliometrix).

## Abbreviations

AC: average citation
AI: artificial intelligence
JMIR: Journal of Medical Internet Research
MCP: multiple-country publication
TC: total citation
TP: total publication
WoSCC: Web of Science Core Collection
