# Supplementary figures and images for: Mapping the Evolution of Digital Health Research: Bibliometric Overview of Research Hotspots, Trends, and Collaboration of Publications in JMIR (1999-2024)
Source: J Med Internet Res. 2024 Oct 17;26:e58987. doi: 10.2196/58987 (PMC11528168; doi:10.2196/58987)

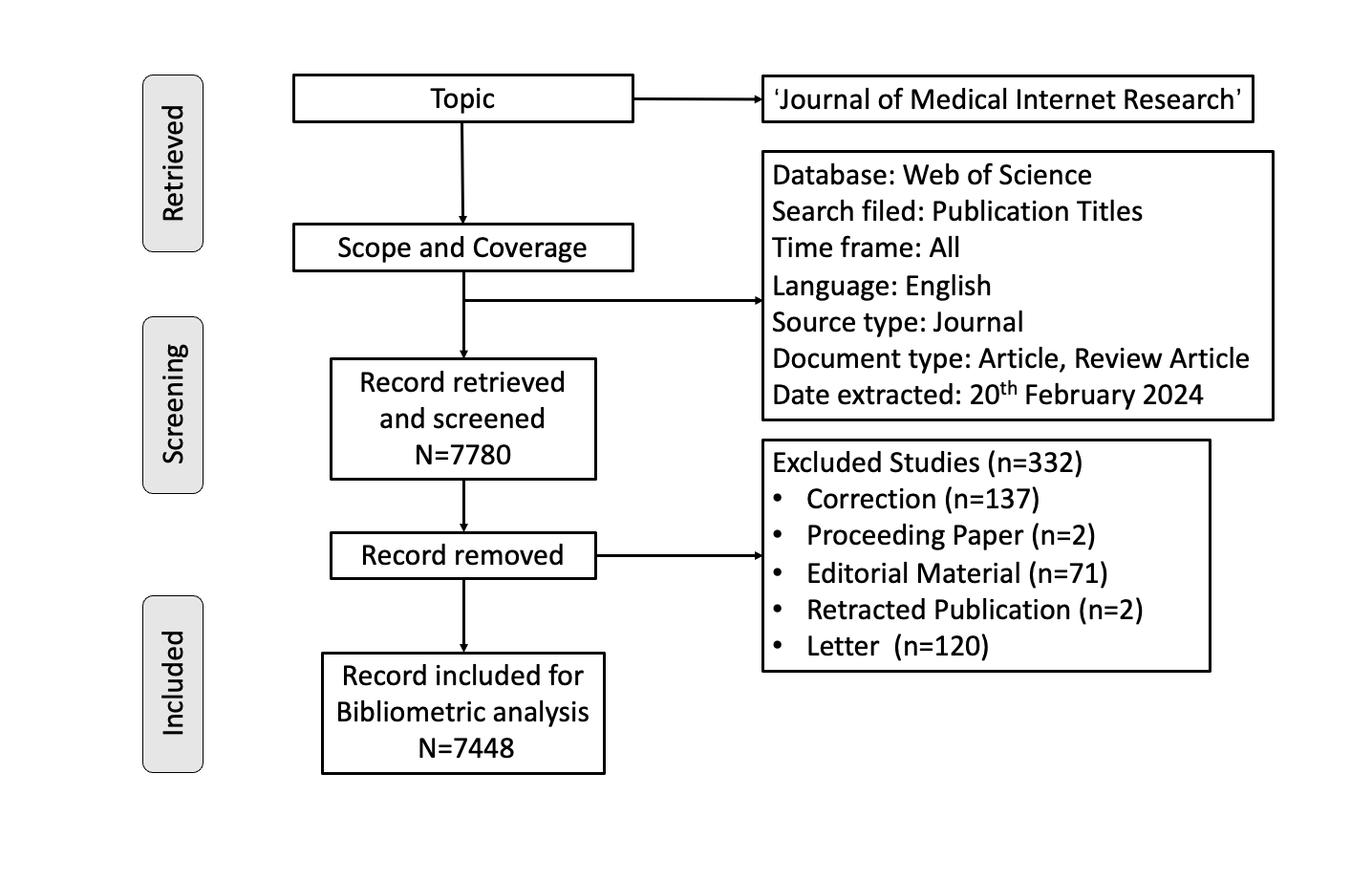

Supplement: Multimedia Appendix 1 [file jmir_v26i1e58987_app1.png]

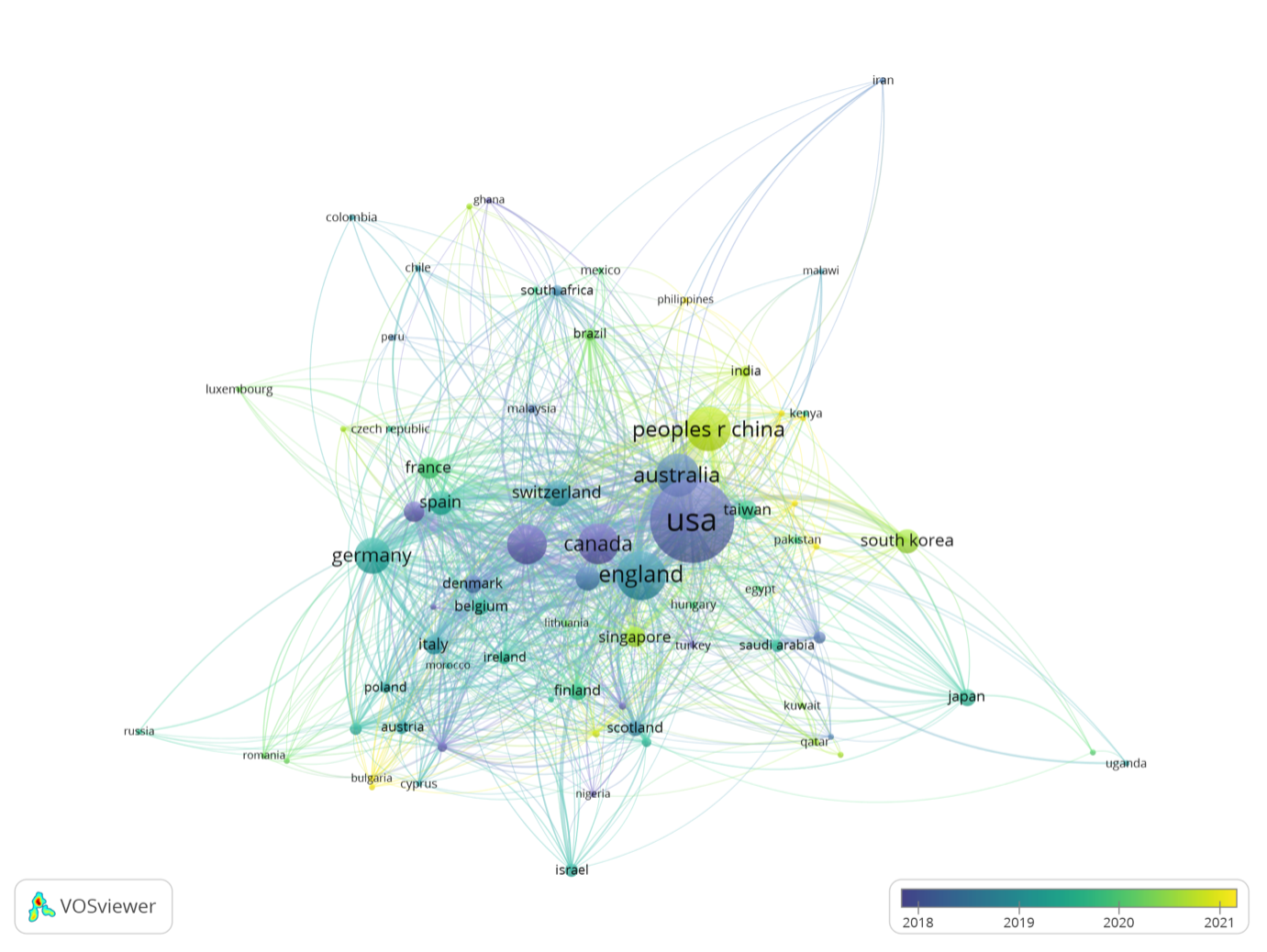

Supplement: Multimedia Appendix 5 [file jmir_v26i1e58987_app5.png]

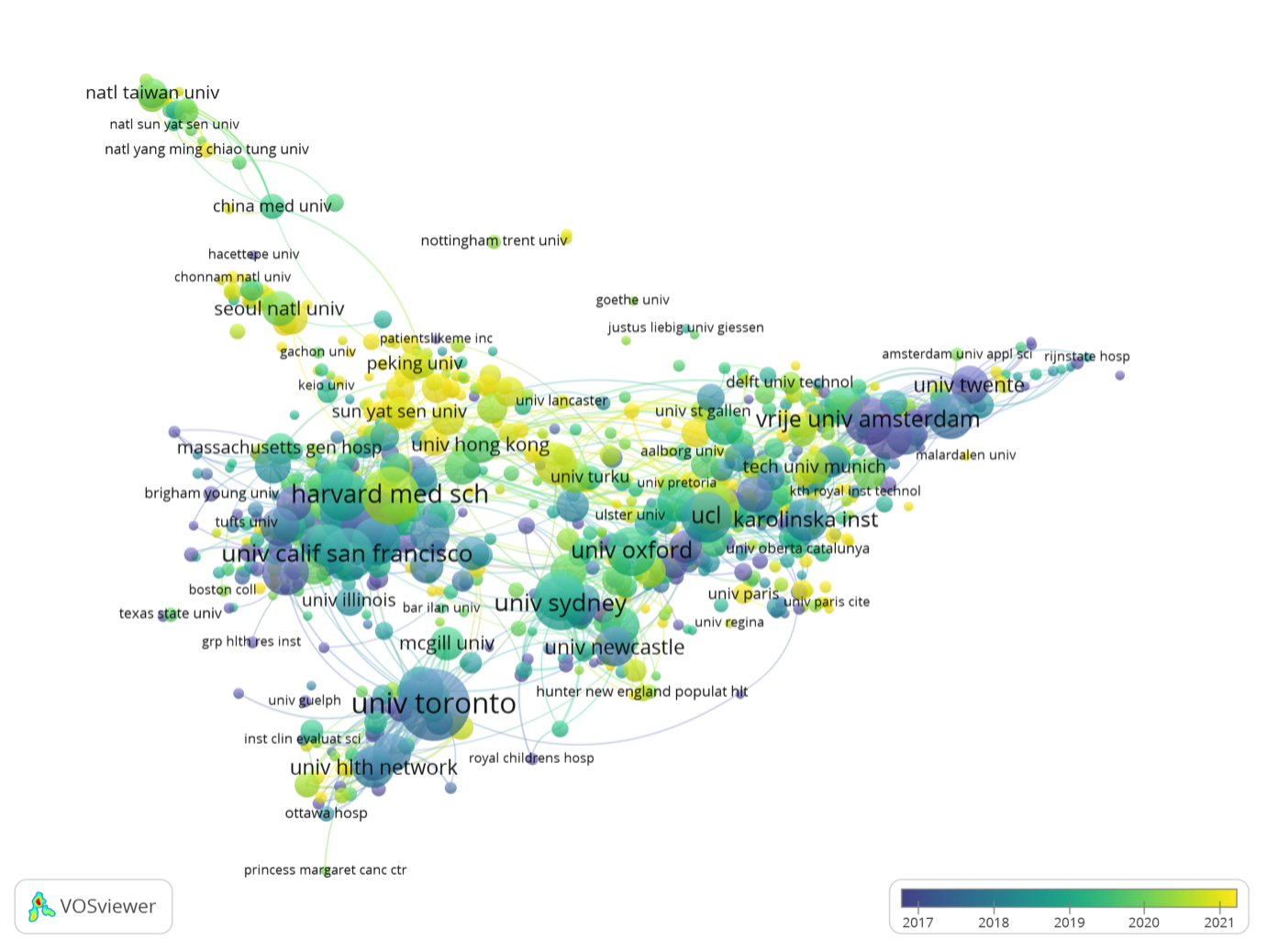

Supplement: Multimedia Appendix 7 [file jmir_v26i1e58987_app7.png]

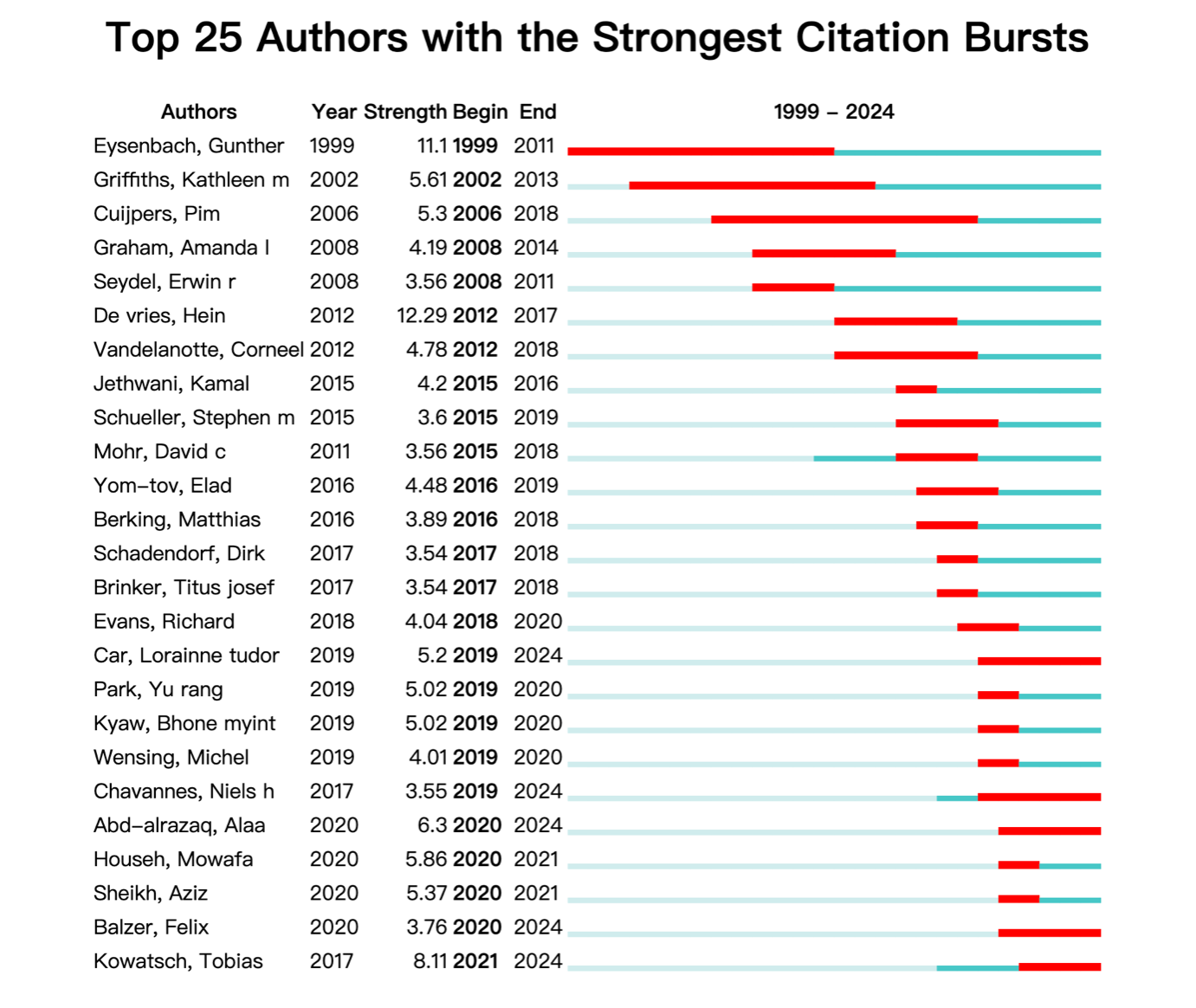

Supplement: Multimedia Appendix 9 [file jmir_v26i1e58987_app9.png]

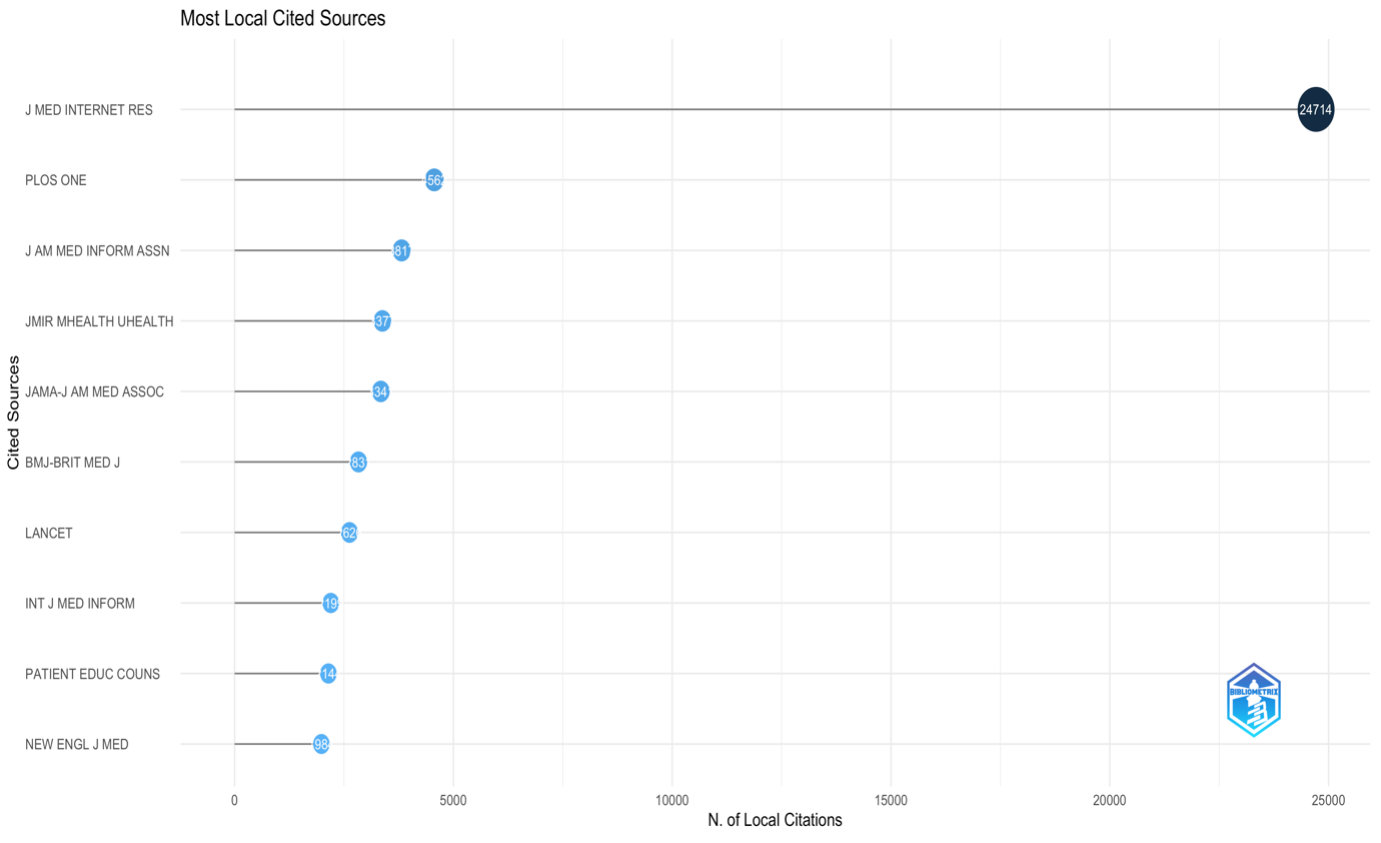

Supplement: Multimedia Appendix 14 [file jmir_v26i1e58987_app14.png]

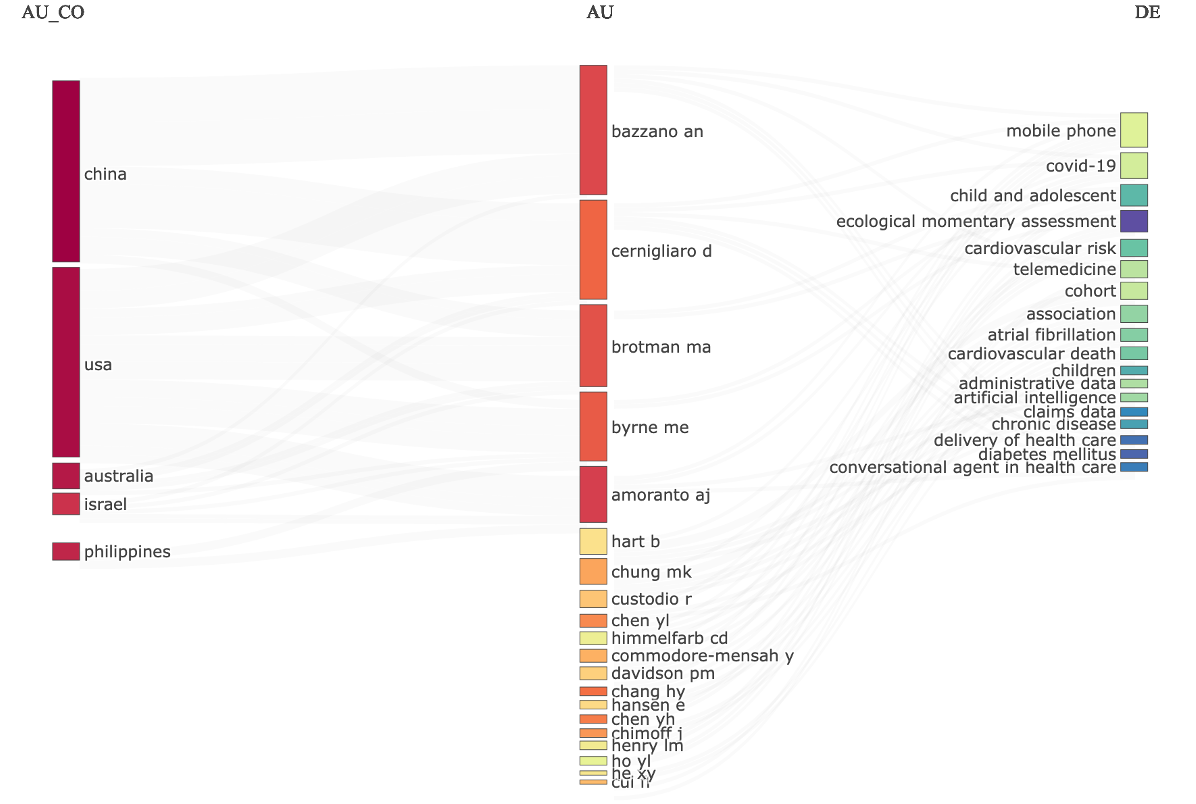

Supplement: Multimedia Appendix 18 [file jmir_v26i1e58987_app18.png]
