# Supplementary material for: Mapping the Evolution of Digital Health Research: Bibliometric Overview of Research Hotspots, Trends, and Collaboration of Publications in JMIR (1999-2024)
Source: J Med Internet Res. 2024 Oct 17;26:e58987. doi: 10.2196/58987 (PMC11528168; doi:10.2196/58987)
Supplement: Multimedia Appendix 2 [file jmir_v26i1e58987_app2.docx]

**Table S1**. A Descriptive Summary of JMIR Publications from 1999 to 2024 (Source from the R package Bibliometrix)

| **Description** | **Results** |
| --- | --- |
| **MAIN INFORMATION ABOUT DATA** | |
| Timespan | 1999:2024 |
| Sources (Journals, Books, etc) | 1 |
| Documents | 7448 |
| Annual Growth Rate % | -0.47 |
| Document Average Age | 5.48 |
| Average citations per doc | 30.29 |
| References | 230270 |
| **DOCUMENT CONTENTS** | |
| Keywords Plus (ID) | 7713 |
| Author's Keywords (DE) | 14337 |
| **AUTHORS** |  |
| Authors | 32232 |
| Authors of single-authored docs | 137 |
| **AUTHORS COLLABORATION** |  |
| Single-authored docs | 158 |
| Co-Authors per Doc | 6.3 |
| International co-authorships % | 28.61 |
| Multiple country publications | 2131.00 |
| Single-country publications | 5317 |
| **DOCUMENT TYPES** |  |
| article | 6306 |
| review | 1142 |
