# Supplementary material for: Mapping the Evolution of Digital Health Research: Bibliometric Overview of Research Hotspots, Trends, and Collaboration of Publications in JMIR (1999-2024)
Source: J Med Internet Res. 2024 Oct 17;26:e58987. doi: 10.2196/58987 (PMC11528168; doi:10.2196/58987)
Supplement: Multimedia Appendix 3 [file jmir_v26i1e58987_app3.docx]

**Table S2. Corresponding Author’s Countries**

| **Country** | **Single Countries Publications (SCP)** | **Multiple Countries Publications  (MCP)** | **Frequency** | **MCP_Ratio*** |
| --- | --- | --- | --- | --- |
| USA | 1830 | 326 | 0.289 | 0.151 |
| UK | 422 | 197 | 0.083 | 0.318 |
| China | 532 | 279 | 0.109 | 0.344 |
| Australia | 409 | 141 | 0.074 | 0.256 |
| Canada | 373 | 124 | 0.067 | 0.249 |
| Netherlands | 335 | 133 | 0.063 | 0.284 |
| Germany | 236 | 148 | 0.052 | 0.385 |
| Switzerland | 81 | 82 | 0.022 | 0.503 |
| Sweden | 109 | 41 | 0.02 | 0.273 |
| Spain | 93 | 61 | 0.021 | 0.396 |

* MCP_Ratio = number of multiple-country publications / total publication, insighting into international collaboration
