# Supplementary material for: Mapping the Evolution of Digital Health Research: Bibliometric Overview of Research Hotspots, Trends, and Collaboration of Publications in JMIR (1999-2024)
Source: J Med Internet Res. 2024 Oct 17;26:e58987. doi: 10.2196/58987 (PMC11528168; doi:10.2196/58987)
Supplement: Multimedia Appendix 4 [file jmir_v26i1e58987_app4.docx]

**Table S3.** Top 25 Countries' Collaboration (Source from the R package Bibliometrix)

| **From** | **To** | **Frequency** |
| --- | --- | --- |
| USA | CHINA | 230 |
| USA | UNITED KINGDOM | 166 |
| USA | CANADA | 146 |
| UNITED KINGDOM | AUSTRALIA | 95 |
| USA | AUSTRALIA | 89 |
| UNITED KINGDOM | NETHERLANDS | 86 |
| CHINA | UNITED KINGDOM | 82 |
| USA | GERMANY | 70 |
| NETHERLANDS | GERMANY | 66 |
| GERMANY | SWITZERLAND | 63 |
| UNITED KINGDOM | GERMANY | 57 |
| USA | SWITZERLAND | 57 |
| UNITED KINGDOM | SWEDEN | 54 |
| UNITED KINGDOM | SWITZERLAND | 54 |
| USA | NETHERLANDS | 54 |
| USA | KOREA | 51 |
| UNITED KINGDOM | SPAIN | 47 |
| UNITED KINGDOM | CANADA | 45 |
| USA | SINGAPORE | 45 |
| UNITED KINGDOM | SINGAPORE | 42 |
| USA | FRANCE | 42 |
| CANADA | AUSTRALIA | 41 |
| UNITED KINGDOM | IRELAND | 41 |
| USA | SPAIN | 41 |
| CHINA | AUSTRALIA | 39 |
