# Supplementary material for: Mapping the Evolution of Digital Health Research: Bibliometric Overview of Research Hotspots, Trends, and Collaboration of Publications in JMIR (1999-2024)
Source: J Med Internet Res. 2024 Oct 17;26:e58987. doi: 10.2196/58987 (PMC11528168; doi:10.2196/58987)
Supplement: Multimedia Appendix 6 [file jmir_v26i1e58987_app6.docx]

**Table S4.** Collaborations among Scholar Institutions (Source from VOSviewer)

| **Organization** | **Country** | **Documents** | **Citations** | **Total Link Strength** |
| --- | --- | --- | --- | --- |
| University of Toronto | Canada | 251 | 12431 | 619 |
| Harvard Medical School | USA | 161 | 3229 | 4887 |
| Harvard University | USA | 96 | 4096 | 304 |
| Imperial College London | UK | 104 | 3285 | 269 |
| University of Melbourne | Australia | 123 | 3278 | 266 |
| Vrije Universiteit Amsterdam | Netherlands | 113 | 6561 | 266 |
| Stanford University | USA | 131 | 4008 | 264 |
| University of California, San Francisco | USA | 133 | 4865 | 249 |
| University College London | UK | 123 | 5839 | 246 |
| University of British Columbia | Canada | 94 | 3396 | 230 |
