# Supplementary material for: Mapping the Evolution of Digital Health Research: Bibliometric Overview of Research Hotspots, Trends, and Collaboration of Publications in JMIR (1999-2024)
Source: J Med Internet Res. 2024 Oct 17;26:e58987. doi: 10.2196/58987 (PMC11528168; doi:10.2196/58987)
Supplement: Multimedia Appendix 8 [file jmir_v26i1e58987_app8.docx]

**Table S5.** The Top 10 Most Frequently Published Authors (Source from WoSCC)

| **Author (Gender)** | **Publications in JMIR** | **Citations** | **Average Citations** | **H-index** | **Institution** | **Country** |
| --- | --- | --- | --- | --- | --- | --- |
| Heleen Riper (F) | 41 | 2469 | 61.46 | 24 | Vrije Universiteit  Amsterdam | Netherlands |
| [Hein de Vries](https://www.heindevries.eu/) (M) | 39 | 1134 | 31.95 | 23 | Maastricht  University | Netherlands |
| Jinseok Lee (M) | 39 | 721 | 18.56 | 15 | Kyung Hee  University | Korea |
| Helen Christensen (F) | 37 | 3751 | 102.41 | 24 | Georgia Institute  of Technology | USA |
| [Pim Cuijpers](https://scholar.google.com/citations?user=N08XHUAAAAAJ&hl=nl) (M) | 30 | 2499 | 84.5 | 22 | Vrije Universiteit  Amsterdam | Netherlands |
| Lorainne Tudor Car (F) | 27 | 1346 | 52.48 | 17 | Nanyang Technological  University (NTU) Singapore | Singapore |
| Josip Car (M) | 25 | 871 | 36.48 | 13 | Nanyang Technological  University (NTU) Singapore | Singapore |
| Yan Zhang (F) | 25 | 505 | 20.32 | 13 | Texas Christian  University | USA |
| David Daniel Ebert (M) | 24 | 1280 | 54.79 | 14 | GETON Inst Online  GesundheitsTrainings GmbH | Germany. |
| Tobias Kowatsch (M) | 24 | 450 | 19.79 | 10 | University of  Zurich | Switzerland |
