# Supplementary material for: Mapping the Evolution of Digital Health Research: Bibliometric Overview of Research Hotspots, Trends, and Collaboration of Publications in JMIR (1999-2024)
Source: J Med Internet Res. 2024 Oct 17;26:e58987. doi: 10.2196/58987 (PMC11528168; doi:10.2196/58987)
Supplement: Multimedia Appendix 10 [file jmir_v26i1e58987_app10.docx]

**Table S6.** The Top 10 Most Frequently Collaborated Authors (Source from VOSviewer)

| **Author (Gender)** | **Documents** | **Citation** | **Total link strength** |
| --- | --- | --- | --- |
| [Heleen Riper](https://psychiatryamsterdam.nl/heleen-riper/) (F) | 40 | 2477 | 100 |
| [Hein de Vries](https://www.heindevries.eu/) (M) | 39 | 1237 | 81 |
| [Lorainne Tudor Car](https://scholar.google.com/citations?user=HMfBYr4AAAAJ&hl=en) (F) | 27 | 1381 | 76 |
| [Pim Cuijpers](https://scholar.google.com/citations?user=N08XHUAAAAAJ&hl=nl) (M) | 30 | 2511 | 72 |
| [David Daniel Ebert](https://www.professoren.tum.de/en/ebert-david-daniel) (M) | 21 | 1266 | 68 |
| Helen Christensen (F) | 31 | 3420 | 64 |
| [Matthias Berking](https://en.wikipedia.org/wiki/Matthias_Berking) (M) | 14 | 925 | 55 |
| [Josip Car](https://www.kcl.ac.uk/people/josip-car) (M) | 24 | 889 | 55 |
| [Luke Wolfenden](https://www.newcastle.edu.au/profile/luke-wolfenden) (M) | 12 | 122 | 49 |
| [Dirk Lehr](https://scholar.google.de/citations?user=boalxaQAAAAJ&hl=de) (M) | 14 | 797 | 47 |
