# Supplementary material for: Mapping the Evolution of Digital Health Research: Bibliometric Overview of Research Hotspots, Trends, and Collaboration of Publications in JMIR (1999-2024)
Source: J Med Internet Res. 2024 Oct 17;26:e58987. doi: 10.2196/58987 (PMC11528168; doi:10.2196/58987)
Supplement: Multimedia Appendix 11 [file jmir_v26i1e58987_app11.docx]

**Table S7.** The Top 10 Influential Authors Among Co-Citation Analysis (Source from VOSviewer)

| **Author (Gender)** | **Citations** | **Total link strength** |
| --- | --- | --- |
| Gunther Eysenbach (M) | 1857 | 7626 |
| Helen Christensen (F) | 392 | 4165 |
| [Gerhard Andersson](https://scholar.google.com/citations?user=ilKS0fUAAAAJ&hl=en) (M) | 301 | 3566 |
| David C Mohr (M) | 361 | 3413 |
| [Nickolai Titov](https://pubmed.ncbi.nlm.nih.gov/?size=200&term=Titov+N&cauthor_id=21679925) (M) | 209 | 3141 |
| Russell E. Glasgow (M) | 384 | 3083 |
| [Pim Cuijpers](https://scholar.google.com/citations?user=N08XHUAAAAAJ&hl=nl) (M) | 273 | 3077 |
| [Susan Michie](https://en.wikipedia.org/wiki/Susan_Michie) (F) | 456 | 2582 |
| Albert Bandura (M) | 400 | 2410 |
| Saskia M. Kelders (F) | 243 | 2325 |
