# Supplementary material for: Mapping the Evolution of Digital Health Research: Bibliometric Overview of Research Hotspots, Trends, and Collaboration of Publications in JMIR (1999-2024)
Source: J Med Internet Res. 2024 Oct 17;26:e58987. doi: 10.2196/58987 (PMC11528168; doi:10.2196/58987)
Supplement: Multimedia Appendix 12 [file jmir_v26i1e58987_app12.docx]

**Table S8. Author’s Productivity Through Lotka’s Law***

| **Documents written** | **N. of Authors** | **Proportion of Authors** |
| --- | --- | --- |
| 41 | 1 | <0.001 |
| 40 | 1 | <0.001 |
| 39 | 1 | <0.001 |
| 37 | 1 | <0.001 |
| 30 | 1 | <0.001 |
| 27 | 1 | <0.001 |
| 25 | 2 | <0.001 |
| 24 | 2 | <0.001 |
| 23 | 1 | <0.001 |
| 22 | 5 | <0.001 |
| 21 | 5 | <0.001 |
| 20 | 2 | <0.001 |
| 19 | 3 | <0.001 |
| 18 | 4 | <0.001 |
| 17 | 6 | <0.001 |
| 16 | 9 | <0.001 |
| 15 | 3 | <0.001 |
| 14 | 11 | <0.001 |
| 13 | 7 | <0.001 |
| 12 | 26 | 0.001 |
| 11 | 29 | 0.001 |
| 10 | 25 | 0.001 |
| 9 | 54 | 0.002 |
| 8 | 79 | 0.002 |
| 7 | 95 | 0.003 |
| 6 | 157 | 0.005 |
| 5 | 332 | 0.01 |
| 4 | 641 | 0.02 |
| 3 | 1399 | 0.043 |
| 2 | 4336 | 0.135 |
| 1 | 24993 | 0.775 |

*Lotka’s Law: Proportion of Authors = number of authors published in a specific amount of papers / number of the total contributing authors.
