# Supplementary material for: Mapping the Evolution of Digital Health Research: Bibliometric Overview of Research Hotspots, Trends, and Collaboration of Publications in JMIR (1999-2024)
Source: J Med Internet Res. 2024 Oct 17;26:e58987. doi: 10.2196/58987 (PMC11528168; doi:10.2196/58987)
Supplement: Multimedia Appendix 13 [file jmir_v26i1e58987_app13.docx]

**Table S9.** The Top 25 Most Citing Sources by the JMIR Authors (Source from WoSCC)

| **Journal** | **Articles** | **Percentage** |
| --- | --- | --- |
| JOURNAL OF MEDICAL INTERNET RESEARCH | 5,982 | 4.89% |
| INTERNATIONAL JOURNAL OF ENVIRONMENTAL RESEARCH AND PUBLIC HEALTH | 2,538 | 2.08% |
| PLOS ONE | 1,915 | 1.57% |
| JMIR MHEALTH AND UHEALTH | 1,641 | 1.34% |
| BMJ OPEN | 1,472 | 1.20% |
| JMIR FORMATIVE RESEARCH | 1,377 | 1.13% |
| FRONTIERS IN PUBLIC HEALTH | 1,250 | 1.02% |
| BMC PUBLIC HEALTH | 1,029 | 0.84% |
| FRONTIERS IN PSYCHOLOGY | 911 | 0.75% |
| HEALTHCARE | 780 | 0.64% |
| JMIR RESEARCH PROTOCOLS | 759 | 0.62% |
| DIGITAL HEALTH | 708 | 0.58% |
| INTERNATIONAL JOURNAL OF MEDICAL INFORMATICS | 703 | 0.58% |
| FRONTIERS IN PSYCHIATRY | 697 | 0.57% |
| BMC HEALTH SERVICES RESEARCH | 648 | 0.53% |
| JMIR MENTAL HEALTH | 606 | 0.50% |
| STUDIES IN HEALTH TECHNOLOGY AND INFORMATICS | 598 | 0.49% |
| TELEMEDICINE AND E HEALTH | 598 | 0.49% |
| SCIENTIFIC REPORTS | 595 | 0.49% |
| JOURNAL OF THE AMERICAN MEDICAL INFORMATICS ASSOCIATION | 586 | 0.48% |
| BMC MEDICAL INFORMATICS AND DECISION MAKING | 537 | 0.44% |
| PATIENT EDUCATION AND COUNSELING | 518 | 0.42% |
| LECTURE NOTES IN COMPUTER SCIENCE | 514 | 0.42% |
| SENSORS | 501 | 0.41% |
| NUTRIENTS | 493 | 0.40% |
