# Supplementary material for: Mapping the Evolution of Digital Health Research: Bibliometric Overview of Research Hotspots, Trends, and Collaboration of Publications in JMIR (1999-2024)
Source: J Med Internet Res. 2024 Oct 17;26:e58987. doi: 10.2196/58987 (PMC11528168; doi:10.2196/58987)
Supplement: Multimedia Appendix 15 [file jmir_v26i1e58987_app15.docx]

**Table S10. The Top 10 Most Local Cited Documents**

| **Title** | **Author** | **Year** | **Local Citations** | **Global Citations** | **LC/GC Ratio (%)** | **Normalized Local Citations** | **Normalized Global Citations** |
| --- | --- | --- | --- | --- | --- | --- | --- |
| The Law of Attrition [22] | EYSENBACH G | 2005 | 326 | 1607 | 20.29 | 22.41 | 15.88 |
| Using the Internet to Promote Health Behavior Change: A Systematic Review and Meta-analysis of the Impact of Theoretical Basis, Use of Behavior Change Techniques, and Mode of Delivery on Efficacy [17] | WEBB TL | 2010 | 192 | 1575 | 12.19 | 15.66 | 16.87 |
| Persuasive System Design Does Matter: A Systematic Review of Adherence to Web-Based Interventions [18] | KELDERS SM | 2012 | 149 | 758 | 19.66 | 18.09 | 11.45 |
| eHealth Literacy: Essential Skills for Consumer Health in a Networked World [26] | NORMAN CD | 2006 | 136 | 1228 | 11.07 | 7.00 | 7.38 |
| Adherence in Internet Interventions for Anxiety and Depression: Systematic Review [19] | CHRISTENSEN H | 2009 | 130 | 747 | 17.40 | 9.75 | 7.08 |
| A Systematic Review of the Impact of Adherence on the Effectiveness of e-Therapies[1] | DONKIN L | 2011 | 111 | 526 | 21.10 | 9.19 | 6.15 |
| A New Dimension of Health Care: Systematic Review of the Uses, Benefits, and Limitations of Social Media for Health Communication [25] | MOORHEAD SA | 2013 | 106 | 1259 | 8.42 | 14.90 | 18.20 |
| Patient Portals and Patient Engagement: A State of the Science Review[2] | IRIZARRY T | 2015 | 87 | 451 | 19.29 | 13.66 | 7.33 |
| A Holistic Framework to Improve the Uptake and Impact of eHealth Technologies[3] | VAN GEMERT-PIJNEN JEWC | 2011 | 84 | 552 | 15.22 | 6.95 | 6.45 |
| Which Intervention Characteristics are Related to More Exposure to Internet-Delivered Healthy Lifestyle Promotion Interventions? A Systematic Review[4] | BROUWER W | 2011 | 84 | 314 | 26.75 | 6.95 | 3.67 |

## References

1. Donkin L, Christensen H, Naismith SL, Neal B, Hickie IB, Glozier N. A systematic review of the impact of adherence on the effectiveness of e-therapies. J Med Internet Res. 2011 Aug 5;13(3):e52. PMID: 21821503. doi: 10.2196/jmir.1772.

2. Irizarry T, DeVito Dabbs A, Curran CR. Patient Portals and Patient Engagement: A State of the Science Review. J Med Internet Res. 2015 Jun 23;17(6):e148. PMID: 26104044. doi: 10.2196/jmir.4255.

3. van Gemert-Pijnen JE, Nijland N, van Limburg M, Ossebaard HC, Kelders SM, Eysenbach G, et al. A holistic framework to improve the uptake and impact of eHealth technologies. J Med Internet Res. 2011 Dec 5;13(4):e111. PMID: 22155738. doi: 10.2196/jmir.1672.

4. Brouwer W, Kroeze W, Crutzen R, de Nooijer J, de Vries NK, Brug J, et al. Which intervention characteristics are related to more exposure to internet-delivered healthy lifestyle promotion interventions? A systematic review. J Med Internet Res. 2011 Jan 6;13(1):e2. PMID: 21212045. doi: 10.2196/jmir.1639.
