# Supplementary material for: Mapping the Evolution of Digital Health Research: Bibliometric Overview of Research Hotspots, Trends, and Collaboration of Publications in JMIR (1999-2024)
Source: J Med Internet Res. 2024 Oct 17;26:e58987. doi: 10.2196/58987 (PMC11528168; doi:10.2196/58987)
Supplement: Multimedia Appendix 16 [file jmir_v26i1e58987_app16.docx]

**Table S11. The top 10 Citing Reference by JMIR Authors**

| **Title** | **Journal** | **First Author** | **Published Year** | **Country** | **Citations by JMIR** |
| --- | --- | --- | --- | --- | --- |
| Preferred Reporting Items for Systematic Reviews and Meta-Analyses: The PRISMA Statement [1] | INTERNATIONAL JOURNAL OF SOURGERY / THE BMJ | David Moher | 2009 | Canada | 331 |
| The Law of Attrition [22] | JOURNAL OF MEDICAL INTERNET RESEARCH | Gunther Eysenbach | 2005 | Canada | 326 |
| CONSORT-EHEALTH: Improving and Standardizing Evaluation Reports of Web-based and Mobile Health Interventions [2] | JOURNAL OF MEDICAL INTERNET RESEARCH | Gunther Eysenbach | 2012 | Canada | 310 |
| Using thematic analysis in psychology [3] | QUALITATIVE RESEARCH IN PSYCHOLOGY | Virginia Braun | 2006 | New Zealand | 254 |
| Using the Internet to Promote Health Behavior Change: A Systematic Review and Meta-analysis of the Impact of Theoretical Basis, Use of Behavior Change Techniques, and Mode of Delivery on Efficacy [17] | JOURNAL OF MEDICAL INTERNET RESEARCH | Thomas Webb | 2010 | UK | 192 |
| GPOWER: A general power analysis program [4] | BEHAVIOR RESEARCH METHODS, INSTRUMENTS, & COMPUTERS | Edgar Erdfelder | 1996 | Germany | 172 |
| The PHQ-9: validity of a brief depression severity measure [5] | JOURNAL OF GENERAL INTERNAL MEDICINE | Kurt Kroenke | 2001 | Germany | 155 |
| PRISMA extension for scoping reviews (PRISMA-ScR): checklist and explanation [6] | ANNALS OF INTERNAL MEDICINE | Andrea Tricco | 2018 | Canada | 153 |
| Persuasive System Design Does Matter: A Systematic Review of Adherence to Web-Based Interventions [18] | JOURNAL OF MEDICAL INTERNET RESEARCH | Saskia M Kelders | 2012 | Netherlands | 149 |
| Infodemiology and Infoveillance: Framework for an Emerging Set of Public Health Informatics Methods to Analyze Search, Communication and Publication Behavior on the Internet [7] | JOURNAL OF MEDICAL INTERNET RESEARCH | Gunther Eysenbach | 2009 | Canada | 138 |

## References

1. Moher D, Liberati A, Tetzlaff J, Altman DG, Group P. Preferred reporting items for systematic reviews and meta-analyses: the PRISMA statement. PLoS Med. 2009 Jul 21;6(7):e1000097. PMID: 19621072. doi: 10.1371/journal.pmed.1000097.

2. Eysenbach G, Group C-E. CONSORT-EHEALTH: improving and standardizing evaluation reports of Web-based and mobile health interventions. J Med Internet Res. 2011 Dec 31;13(4):e126. PMID: 22209829. doi: 10.2196/jmir.1923.

3. Braun V, Clarke V. Using thematic analysis in psychology. Qualitative Research in Psychology. 2006 01/01;3:77-101. doi: 10.1191/1478088706qp063oa.

4. Erdfelder E, Faul F, Buchner A. GPOWER: A general power analysis program. Behavior Research Methods, Instruments, & Computers. 1996 1996/03/01;28(1):1-11. doi: 10.3758/BF03203630.

5. Kroenke K, Spitzer RL, Williams JB. The PHQ-9: validity of a brief depression severity measure. J Gen Intern Med. 2001 Sep;16(9):606-13. PMID: 11556941. doi: 10.1046/j.1525-1497.2001.016009606.x.

6. Tricco AC, Lillie E, Zarin W, O'Brien KK, Colquhoun H, Levac D, et al. PRISMA Extension for Scoping Reviews (PRISMA-ScR): Checklist and Explanation. Ann Intern Med. 2018 Oct 2;169(7):467-73. PMID: 30178033. doi: 10.7326/m18-0850.

7. Eysenbach G. Infodemiology and infoveillance: framework for an emerging set of public health informatics methods to analyze search, communication and publication behavior on the Internet. J Med Internet Res. 2009 Mar 27;11(1):e11. PMID: 19329408. doi: 10.2196/jmir.1157.
