# Supplementary material for: Mapping the Evolution of Digital Health Research: Bibliometric Overview of Research Hotspots, Trends, and Collaboration of Publications in JMIR (1999-2024)
Source: J Med Internet Res. 2024 Oct 17;26:e58987. doi: 10.2196/58987 (PMC11528168; doi:10.2196/58987)
Supplement: Multimedia Appendix 17 [file jmir_v26i1e58987_app17.docx]

**Table S12.** Summary of 11 Clusters Analysis for Keywords (Source from CiteSpace)

| **Cluster ID** | **Cluster Name** | **Size** | **Silhouette Value** | **Mean Year** | **Label (LLR)** | **Label (LSI)** |
| --- | --- | --- | --- | --- | --- | --- |
| 0 | Electronic health records | 321 | 0.566 | 2009 | electronic health records (129.2, 1.0E-4); patient portals (113.41, 1.0E-4); patient portal (112.11, 1.0E-4); blockchain (94.81, 1.0E-4); qualitative research (91.76, 1.0E-4) | health care; hospital systems; digital adherence technologies; general practitioner; information technology \| mobile phone; systematic review; hand hygiene compliance; hand hygiene; electronic monitoring systems |
| 1 | Mental health | 181 | 0.67 | 2014 | mental health (243.46, 1.0E-4); depression (237.52, 1.0E-4); anxiety (187.46, 1.0E-4); cognitive behavioral therapy (98.25, 1.0E-4); tobacco (73.51, 1.0E-4) | mental health; digital phenotyping; sensor data; sensing apps; ecological momentary intervention \| mobile phone; behavior change; digital therapeutics; lifestyle intervention; diabetes self-management |
| 2 | Hypertension | 152 | 0.722 | 2017 | hypertension (94.16, 1.0E-4); diabetes (71.79, 1.0E-4); medication adherence (64.13, 1.0E-4); type 2 diabetes mellitus (48.97, 1.0E-4); heart failure (48.28, 1.0E-4) | mobile phone; behavior change; digital interventions; mixed methods; healthy living \| digital health; mental health; digital adherence technologies; information technology; alarm system quality |
| 3 | Internet | 141 | 0.864 | 2008 | internet (118.87, 1.0E-4); randomized controlled trial (104.88, 1.0E-4); twitter (75.45, 1.0E-4); systematic review (72.69, 1.0E-4); natural language processing (63.59, 1.0E-4) | mobile phone; online systems; seeking behavior; information dissemination; evidence-based medicine \| controlled trial; blood pressure; internet technology; vigorous activity; ocular surface disease index |
| 4 | Systematic review | 140 | 0.71 | 2011 | systematic review (344.04, 1.0E-4); meta-analysis (168.38, 1.0E-4); medical education (167.58, 1.0E-4); scoping review (77.71, 1.0E-4); review (74.64, 1.0E-4) | systematic review; mobile phone; hand hygiene; hand hygiene compliance; hand hygiene quality \| digital health; conversational agent; digital health companies; digital behavior change intervention; in-time adaptive intervention |
| 5 | Machine learning | 129 | 0.804 | 2017 | machine learning (597.46, 1.0E-4); artificial intelligence (282.78, 1.0E-4); deep learning (253.21, 1.0E-4); internet (119.32, 1.0E-4); natural language processing (106.47, 1.0E-4) | machine learning; blood glucose dynamics; anomalies detection; coronary artery disease; language models \| artificial intelligence; atrial fibrillation; heart rhythm; software algorithm; fibrillation detection |
| 6 | Dementia | 104 | 0.717 | 2016 | dementia (183.53, 1.0E-4); reliability (89.51, 1.0E-4); mild cognitive impairment (84.4, 1.0E-4); Alzheimer disease (82.41,1.0E-4); older adults (80.31, 1.0E-4) | mobile phone; health literacy; questionnaire design; health equity; validity evidence \| digital health; mobile health; chronic disease; multiple chronic conditions; data collection methods |
| 7 | Information seeking behavior | 102 | 0.746 | 2013 | information seeking behavior (105.88, 1.0E-4); digital divide (89.79, 1.0E-4); health literacy (71.14, 1.0E-4); consumer health information (64.11, 1.0E-4); health disparities (61.13, 1.0E-4) | mobile phone; digital divide; socioeconomic factors; distance counseling; psychotic disorders \| social media; sexual health; mobile applications; online social networking; social norms |
| 8 | Covid-19 | 100 | 0.841 | 2017 | covid-19 (918.3, 1.0E-4); pandemic (224.9, 1.0E-4); public health (184.94, 1.0E4); infodemiology (169.9, 1.0E-4); mobile phone (151.7, 1.0E-4) | social media; public health; infectious disease; sentiment analysis; emotion analysis \| digital health; google trends; real-world data; seeking behaviour; general practitioner |
| 9 | Hiv | 91 | 0.794 | 2015 | hiv (207.6, 1.0E-4); men who have sex with men (160.86, 1.0E-4); social networks (95.91, 1.0E-4); msm (89.91, 1.0E-4); sexual health (85.84, 1.0E-4) | social media; social network analysis; public engagement; medical research; digital adherence technologies \| mobile phone; substance use; sexual risk reduction; prevention programs; web-based intervention |
| 10 | Physical activity | 78 | 0.807 | 2013 | physical activity (403.04, 1.0E-4); weight loss (195.34, 1.0E-4); obesity (192.36, 1.0E-4); exercise (145, 1.0E-4); behavior change (117.78, 1.0E-4) | physical activity; mobile phone; behavior change; financial incentives; deposit contracts \| health behavior; health promotion; social media; public health; physical education |
| 11 | Parents | 29 | 0.925 | 2014 | parents (129.54, 1.0E-4); child (105.21, 1.0E-4); parenting (88.1, 1.0E-4); infant (80.88, 1.0E-4); children (72.66, 1.0E-4) | mobile phone; physical activity; clinical decision support system; text analysis; web-based questionnaire \| social media; health information; web-based resources; search engine; coping behavior |
